# Two Aquaporin Genes, *GhPIP2;7* and *GhTIP2;1*, Positively Regulate the Tolerance of Upland Cotton to Salt and Osmotic Stresses

**DOI:** 10.3389/fpls.2021.780486

**Published:** 2022-02-11

**Authors:** Anhui Guo, Jianfeng Hao, Ying Su, Bin Li, Nan Zhao, Meng Zhu, Yi Huang, Baoming Tian, Gongyao Shi, Jinping Hua

**Affiliations:** ^1^Laboratory of Cotton Genetics, Genomics and Breeding, Beijing Key Laboratory of Crop Genetic Improvement, Key Laboratory of Crop Heterosis and Utilization of Ministry of Education, College of Agronomy and Biotechnology, China Agricultural University, Beijing, China; ^2^Zhengzhou Research Base, State Key Laboratory of Cotton Biology, School of Agricultural Sciences, Zhengzhou University, Zhengzhou, China; ^3^Oil Crops Research Institute, Chinese Academy of Agricultural Sciences, Wuhan, China

**Keywords:** *Gossypium*, aquaporin, gene family, salt stress, osmotic stress

## Abstract

Aquaporins (AQPs) facilitate the transport of water and small molecules across intrinsic membranes and play a critical role in abiotic stresses. In this study, 111, 54, and 56 candidate *AQP* genes were identified in *Gossypium hirsutum* (AD_1_), *Gossypium arboreum* (A_2_), and *Gossypium raimondii* (D_5_), respectively, and were further classified into five subfamilies, namely, plasma intrinsic protein (PIP), tonoplast intrinsic protein (TIP), nodulin 26-like intrinsic protein (NIP), small basic intrinsic protein (SIP), and uncategorized X intrinsic protein (XIP). Transcriptome analysis and quantitative real-time PCR (qRT-PCR) revealed some high-expression *GhPIPs* and *GhTIPs* (PIP and TIP genes in *G. hirsutum*, respectively) in drought and salt stresses. *GhPIP2;7*-silenced plants decreased in the chlorophyll content, superoxide dismutase (SOD) activity, and peroxidase (POD) activity comparing the mock control (empty-vector) under 400 mM NaCl treatment, which indicated a positive regulatory role of *GhPIP2;7* in salt tolerance of cotton. The *GhTIP2;1*-silenced cotton plants were more sensitive to osmotic stress. *GhTIP2;1*-overexpressed plants exhibited less accumulation of H_2_O_2_ and malondialdehyde but higher proline content under osmotic stress. In summary, our study elucidates the positive regulatory roles of two *GhAQPs* (*GhPIP2;7* and *GhTIP2;1*) in salt and osmotic stress responses, respectively, and provides a new gene resource for future research.

## Introduction

Aquaporins (AQPs) are members of the major intrinsic protein (MIP) superfamily, contributing to the transport of water and small molecules across biological membranes in most organisms (Maurel et al., 2015). Plant AQPs are multifunctional channels with a wide range of selectivity profiles (Baiges et al., 2002). Additionally, AQPs are involved in the transportation of glycerol, urea, ammonia (NH_3_), carbon dioxide (CO_2_), hydrogen peroxide (H_2_O_2_), as well as metalloid such as boron and silicon (Tyerman et al., 2021). AQP family is characterized with six transmembrane domains (TM1–TM6) connected by five loops (LA–LE), two Asn-Pro-Ala (NPA) motifs, aromatic/arginine (ar/R) filter, and Froger’s position (Kaldenhoff and Fischer, 2006). Generally, AQPs are divided into five subfamilies, including plasma intrinsic proteins (PIPs), tonoplast intrinsic proteins (TIPs), nodulin 26-like intrinsic proteins (NIPs), small basic intrinsic proteins (SIPs), and uncategorized X intrinsic proteins (XIPs) (Johanson et al., 2001). At present, 35, 47, 41, 45, 43, 33, 47, and 35 AQPs have been identified in *Arabidopsis* (Johanson et al., 2001), *Solanum lycopersicum* (Reuscher et al., 2013), *Phaseolus vulgaris* (Ariani and Gepts, 2015), *Manihot esculenta* (Putpeerawit et al., 2017), *Zea mays* (Chaumont et al., 2001), *Oryza sativa* (Sakurai et al., 2005), banana (Hu et al., 2015), and watermelon (Zhou et al., 2019), respectively.

Upland cotton (*Gossypium hirsutum*), which is one of the cultivated tetraploid species (2*n* = 52), provides the most common natural textile fibers (Zhang et al., 2015). So far, the complete genome sequences of *G. hirsutum* (AD_1_) TM-1, *Gossypium arboreum* (A_2_) *Shixiya 1*, and *Gossypium raimondii* (D_5_) have been released (Wang et al., 2012; Li F. et al., 2014; Zhang et al., 2015; Chen et al., 2020). *G. hirsutum* is constituted by A subgenome (A*t*) and D subgenome (D*t*), as a result of interspecific hybridization between the progenitors of A-genome resembling *G. arboreum* and D-genome resembling *G. raimondii* (Senchina, 2003; Chen et al., 2017). The accessibility of genomic data has boosted the identification and function research of the *AQP* genes in cotton species.

Crops suffer a significant reduction in quality and production under various abiotic stresses, including drought and salinity stresses (Chen et al., 2021). The main signal caused by drought is osmotic stress. Salt stress caused the imbalance of cellular ions, resulting in dehydration, osmotic stress, and ion toxicity (Zelm et al., 2020). The pathways activated by drought and salt stress are overlapped to a certain degree (Zhu, 2016), in which *AQP* and ion carrier genes involved in signaling cascades and transcriptional regulation are activated to protect the membranes and proteins by controlling the uptake and transport of water and ions (Gong et al., 2020; Gong, 2021). AQPs in different subfamilies show varying expression patterns in response to salt and drought stresses (Zargar et al., 2017). PIPs and TIPs maintained cell water balance as water transporters in *Arabidopsis* (Wang et al., 2019). The overexpression (OE) of PIPs and TIPs increased drought tolerance by decreasing transpiration rate and stomatal conductance (Pou et al., 2013). The root hydraulic conductivity increased in *PIP2;7-*overexpressed plants (Pou et al., 2016). *MaPIP1;1* improved the salt and drought tolerances by regulating primary root elongation, water uptaking, and membrane stability in *Arabidopsis* (Xu et al., 2014). In maize, the expression of three specific isoforms (*ZmPIP1;1*, *ZmPIP1;5*, and *ZmPIP2;4*) was transiently induced when plants regained the osmotic potential for water uptake (Zhu et al., 2005). *TsTIP1;2* protects *Thellungiella salsuginea* from salinity stress by mediating the conduction of H_2_O_2_ and H_2_O across the membrane (Wang et al., 2014). These studies suggest that AQPs have an important role in response to drought and salt stresses in diverse plant species. Although previous studies have identified the gene structure, the phylogenetic relationship of AQPs in upland cotton (Park et al., 2010; Li W. et al., 2019) and the mode of AQPs in the stress response of upland cotton remain largely unknown.

Our previous research identified several AQPs that were involved in the salt stress response of cotton through RNA-seq analysis (Shi et al., 2015). Furthermore, we analyzed the conserved motifs, chromosomal distribution, and gene duplication of *AQP* genes in *G. hirsutum*, *G. arboreum*, and *G. raimondii* and assessed the expression patterns of *AQP*s under salt and drought stress in *G. hirsutum.* A total of three salt stress genes (*GhPIP2;2*, *GhPIP2;3*, and *GhPIP2;7*) and four drought stress genes (*GhPIP1;2*, *GhPIP2;3*, *GhTIP1;1*, and *GhTIP2;1*) were selected for further analysis, such as virus-induced gene silencing (VIGS) in upland cotton and OE in *Arabidopsis.* The transcriptional levels of salt stress-related genes were compared between the *GhTIP2;1-*overexpressed lines and the wild type (WT) under salt and drought treatments. These results provide genetic evidence for the roles of *AQP* genes in plant responses to abiotic stresses.

## Materials and Methods

### Plant Materials

The *G. hirsutum* cultivar GX100-2 was used for qRT-PCR and salt tolerance assay, and Zhong79 was used for qRT-PCR and drought tolerance assay. The 5-day-old cotton seedlings with the same growth state were transferred into the Hoagland liquid medium (Zhang et al., 2011) which was continuously aerated at a temperature regime of 28/20°C with 16-h light/8-h dark cycle. At the trefoil stage of the seedlings, the expression profile of candidate genes was determined by treating half of the seedlings with salt (150 mM NaCl) and the remaining half with deionized water to serve as the control. The fresh leaves were collected at 0, 1, 3, 12, and 48 h after salt stress (Zhang et al., 2011; Su et al., 2020). These samples were frozen in liquid nitrogen immediately and stored at –80°C for RNA isolation. Three biological repeats in each treatment were performed.

For the VIGS experiment, cotton seeds were soaked overnight in distilled water until the radicle sprouted. Seven sprouted seeds were planted in small pots filled with 1:1 (v/v) of vermiculate and nutritional soil and kept in the greenhouse at 28°C under a 16-h/8-h light/dark photoperiod.

The *Arabidopsis thaliana* Columbia ecotype (Col-0) was used as the WT.

### Expression Profile Analysis

The public expression profile of leaf under salt stress across the time course (0, 1, 3, 6, and 12 h) of *G. hirsutum* TM-1 was obtained from the study by Zhang et al. (2015). The expression data were gene-wise normalized, and the expression patterns were illustrated using the MultiExperiment Viewer (MeV) software.

### RNA Isolation and Quantitative Real-Time PCR

Total RNA was extracted by hexadecyl trimethyl ammonium bromide (CTAB) and precipitated by the ammonium acetate method (Zhao et al., 2012). The cDNA was synthesized using PrimeScript™ RT Reagent Synthesis Kit (TaKaRa, Dalian, China). The gene-specific primer pairs were designed by PrimerPremier software (version 5.0) based on the coding sequences of *GhAQPs* and stress-related genes (Supplementary Table S1). *GhUBQ7* and *AtUBQ* were used as internal references in upland cotton and *Arabidopsis*, respectively (Supplementary Table S1). Gene expression was calculated with the 2^–ΔΔCt^ method (Livak and Schmittgen, 2001). Each sample was analyzed with three technical replicates within each of the three biological duplicates.

### Gene Cloning and Vector Construction

Cotton leaf crumple virus (CLCrV) and tobacco rattle virus (TRV) vectors were used in the VIGS experiment under salt and osmotic stress conditions, respectively. The pCLCrV-fused cDNA fragment of magnesium chelatase subunit I (*GhChlI*) and TRV-fused cDNA fragment of chloroplasts alterados 1 gene (*GhCLA*) were used as a positive control to monitor the efficiency of VIGS experiments. The fragments targeting the candidate genes containing different recognition sites were amplified as a template and integrated into pCLCrVA or TRV2.

For the construction of the 35S:GFP-GhAQP vector, the polymerase chain reaction (PCR) product was ligated into the *Bam*HI site and *Xba*I of the pCAMBIA1300-eGFP vector driven by the cauliflower mosaic virus 35S promoter. This vector was transformed into the *Agrobacterium tumefaciens* strain GV3101.

For the OE study, the 35S:GhTIP2;1 (pCAMBIA1300-GhTIP2;1-eGFP) vector was constructed by digesting the *GhTIP2;1* coding sequence with *Kpn*I and *Xba*I. The digested sequence was then inserted into a pCAMBIA1300 vector fused with green fluorescent protein (GFP) tagging, which contained hygromycin- and kanamycin-resistant genes. This vector was transformed into the *A. tumefaciens* strain GV3101. All the primers used in the vector construction are listed in Supplementary Table S2.

### *Arabidopsis* Transformation

The 35S:GhTIP2;1 vector was transformed into *A. thaliana* (Col-0) by the floral dip method (Clough and Bent, 1998). Positive transformants were selected on the MS medium with 25 mg/L hygromycin and grew until maturation.

### Subcellular Localization and β-Glucuronidase Histochemical Staining

Subcellular localization of AQPs was predicted in WoLFPSORT^1^ (Horton et al., 2007). The 35S:GFP-GhAQP vector was transformed into leaves of tobacco (*Nicotiana benthamiana*) K329 cultivar for the subcellular localization analysis. The signal of GFP was observed under a laser confocal scanning microscope (LSM 880, Zeiss, Germany). The 35S-mCherry-OsTIP1;1 was used as a plant vacuolar maker for the colocalization experiment (Cao et al., 2020). Excitation wavelength used in 488 nm for GFP, and the wavelength range of captured light at 515–555 nm. The excitation wavelength and gain wavelength of mCherry were 561 nm and 580–630 nm, respectively.

To investigate the promoter activity in different tissues, the 8-day-old seedlings that transferred *ProGhTIP2;1*:GUS were used for GUS staining. For stress treatments, the 2-week-old seedlings that were transformed into *ProGhTIP2;1*:GUS were treated in ^1^/_2_ MS medium that was supplemented with or without 10% polyethylene glycol 6000 (PEG6000), 20% PEG6000, and 150 mM NaCl for 24 h. GUS Staining Kit (Biosharp company) was employed for the GUS staining. The samples were immersed in GUS histochemical staining buffers and subsequently incubated at 37°C overnight. The samples were decolorized in 75% ethanol until the color of the negative control plants turned white. GUS activity was estimated based on the presence of blue.

### Virus-Induced Gene Silencing Analysis in Cotton

All constructed vectors were transformed into *A. tumefaciens* strain EHA105 by a heat-shock method. The EHA105 lines contained pCLCrVA, pCLCrVA-genes, and TRV, TRV-genes, vectors were mixed with an equal volume of *A. tumefaciens* containing pCLCrVB and TRV1, respectively, and the mixed solution was used to infiltrate plants. The quantitative real-time PCR (qRT-PCR) was performed to further confirm that candidate genes had been silenced in VIGS experiments. The primers used in the qRT-PCR analysis are listed in Supplementary Table S1, and the primers used in the VIGS experiments are listed in Supplementary Table S2.

The cotyledons of 1-week-old cotton seedlings were infiltrated with the solution containing *A. tumefaciens* of pCLCrV-genes or TRV-genes according to the previous description (Gu et al., 2014; Long et al., 2020). The VIGS experiments were repeated at least three times with more than three individual plants were included.

For salt tolerance assay, plants that infiltrated with a solution containing *A. tumefaciens* of *pCLCrV-GhPIPs* after 10 days were watered by 400 mM NaCl solution regularly after every 4 days until the phenotype appeared (Long et al., 2019).

For drought tolerance assay, plants silencing of GhTIP1;1 (TRV:GhTIP1;1), GhTIP2;1 (TRV:GhTIP2;1), GhPIP1;2 (TRV:GhPIP1;2), and GhPIP2;3 (TRV:GhPIP1;2) were transferred to Hoagland liquid medium containing 13% PEG6000. TRV:GFP with no silencing fragment was used as a control. The phenotype was observed after 13% PEG6000 treatment for 1 week, and the samples of leaves and roots were evaluated for the relative water content (RWC).

### Salt- and Drought-Tolerant Assay in *Arabidopsis*

To identify the stress tolerance of *GhTIP2;1* overexpressed *Arabidopsis* (OE1 and OE3) and WT, seeds were sterilized with 5% (v/v) sodium hypochlorite and cultured on MS media, vernalized for 2 days at 4°C and incubated in a growth room (22°C, 16-h light/8-h dark cycle). Seedlings that had no significant difference in the length of primary roots were transferred to MS media with or without 150 mM NaCl and 15% PEG6000, respectively, for stress analysis. The experiments were carried out with three biological replicates, and each replicate represents 20 seedlings for each line.

### Morphological and Physiological Measurements

To identify the chlorophyll damage caused by salt stress, leaves (infiltrated with a solution containing *A. tumefaciens* of *pCLCrV-GhPIPs* after 10 days) of the same size and from the same position were picked and washed with distilled water and then were floated in salt solution (400 mM NaCl) with the abaxial surface down. Photographs were taken after the phenotype developed. Total chlorophyll content was calculated according to the formula described in the study by Arnon (1949).

The leaves of the gene-silenced plants or seedlings of *Arabidopsis* after NaCl or PEG treatments were used for identifying malondialdehyde (MDA), superoxide dismutase (SOD), peroxidase (POD) activities, H_2_O_2_, and proline content. The MDA content, H_2_O_2_ content, proline content, SOD, and POD activity were determined according to the study by Ullah et al. (2018). The absorbance was measured using a UV-2550 UV-vis spectrophotometer (SHIMADZU). Three biological replications were performed. The enzyme assays were performed in three biological replicates. Proline was extracted and quantified as recommended by Zhao et al. (2009).

### Statistical Analysis

The experiments were conducted with three biological replicates, and each replicate represents at least 12 individuals. Each graphical plot represents the results from three repeats, and the values are displayed as the mean ± SD. Statistical significance was determined using Student’s *t*-tests, and *P*-values < 0.05 were considered statistically significant.

## Results

### Conservation and Differentiation of Aquaporins in *Gossypium*

A total of 54, 56, and 111 candidate *AQP* genes were predicted in *G. arboreum*, *G. raimondii*, and *G. hirsutum*, respectively. The gene structure, conserved motif, and duplicate genes of AQPs in A_2_, D_5_, and AD_1_ were analyzed, which indicated that the structure and properties of AQPs were conserved in each subfamily, yet vary among subfamilies. The physical locations of the *AQP* genes exhibited great diversity and complexity in the genome of *Gossypium*. The *Ka/Ks* values of duplicated *AQPs* were less than 1, suggesting that *AQP* genes have undergone strong purifying selection pressure after segmental duplication and whole-genome duplication (WGD) (Supplementary File).

### Subcellular Localization of GhAQPs

**FIGURE 1:**
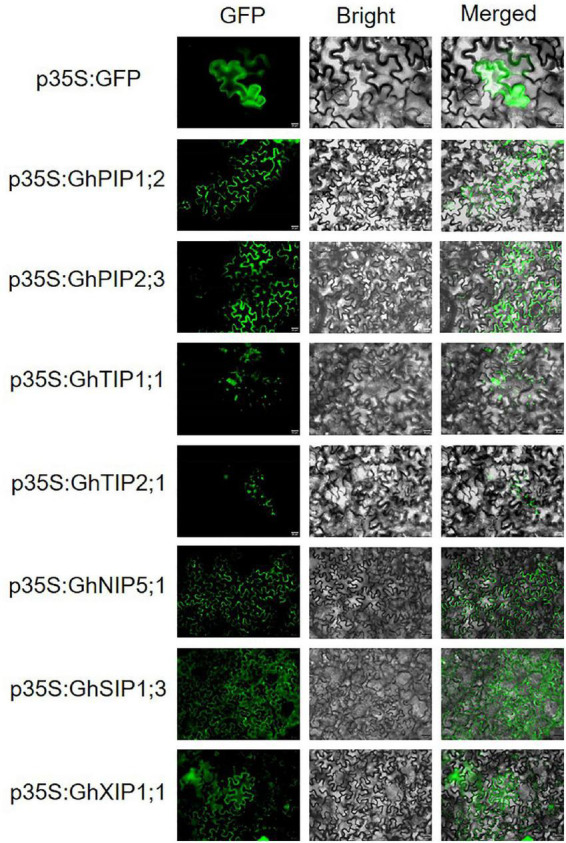
Subcellular localization of GhAQPs. *GhAQPs* were transiently expressed in *Nicotiana benthamiana* to determine its subcellular localization (Bar = 20 μm). GFP, green fluorescent protein.

Most PIPs were predicted to localize on the plasma membrane. TIPs were predicted to localize on the vacuolar membrane and plasma membrane, as well as in the cytoplasm. NIPs, SIPs, and XIPs were predicted to localize on the vacuolar membrane and plasma membrane. To determine the subcellular localization of GhAQP proteins, a C-terminal GFP fusion vector containing GhAQPs driven by a 35S promoter was constructed, and the free GFP vector was used as a positive control. Transient expression in tobacco leaves was performed by agroinfiltration. The 35S:GFP fusion protein was localized in the membrane, cytoplasm, and nucleus (Figure 1). The signals of *GhPIP1;2*, *GhPIP2;3*, and *GhNIP5;1* were perceived in the plasma membrane, yet the signals of *GhTIP1;1* and *GhTIP2;1* were captured in the vacuole membrane. The *GhSIP1;3* and *GhXIP1;1* were localized to the plasma membrane and cytoplasm (Figure 1). The experimental subcellular localization of AQPs was consistent with software prediction.

### Expression Pattern of *GhAQPs* Under Salt and Osmotic Stresses

**FIGURE 2:**
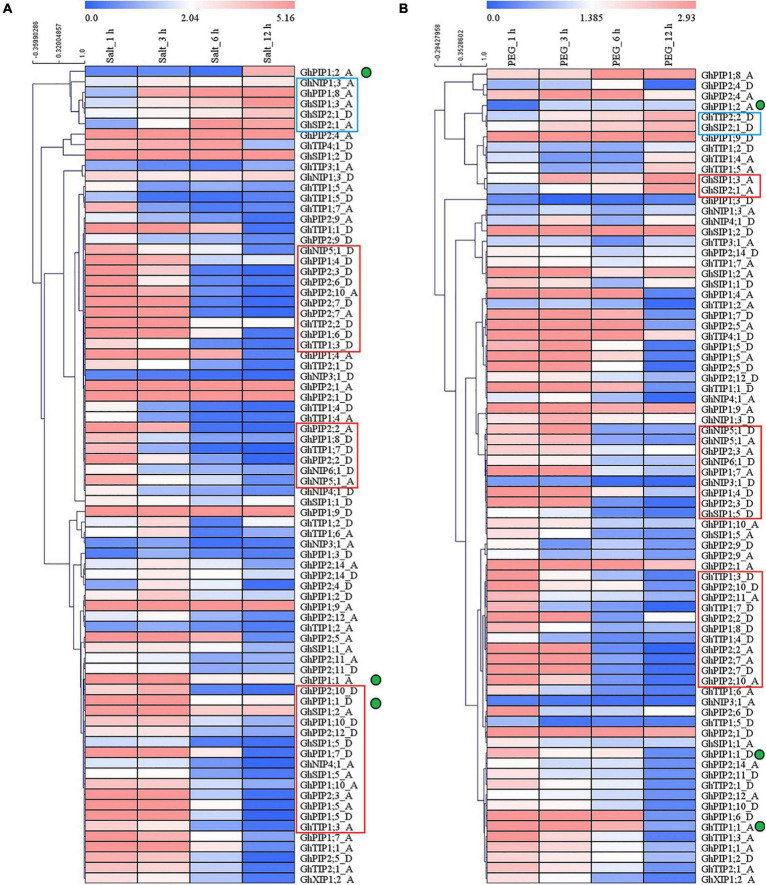
Expression patterns of *GhPIP and GhTIP* genes response to salt and osmotic stresses. The expression patterns of GhAQPs after salt **(A)** and drought stress **(B)**, respectively. The green dots represent the genes with different expression patterns under salt stress and osmotic stress. The red box represents the genes that are highly expressed in the early stage (1–3 h) after stress, and the blue box represents the genes that are highly expressed in the late stage (3–12 h) after stress. The fragments per kilobase of transcript per million mapped reads (FPKM) values of *GhAQP* genes after 200 mM NaCl or PEG treatment were from public RNA-seq data. The fold change values of *GhAQP* genes after salt or drought stresses were shown in the heatmap constructed by the MultiExperiment Viewer (MeV) software.

The expression patterns of homologous *GhAQP* genes in A*t* and D*t* subgenomes were similar under salt or osmotic stress (Figure 2). Most *GhAQPs* (genes in the red box) that were belonging to the PIP subfamily were induced rapidly and continued to be upregulated at 3 h after salt or osmotic treatment.

A few *GhAQPs* (genes in the blue box) were induced at 3 h after salt or osmotic stress and were continuously upregulated until 12 h. Only three gene pairs (*GhPIP1;9_A/D*, *GhPIP2;1_A/D*, and *GhSIP1;2_A/D*) were continuously upregulated after salt and osmotic stresses. The expression patterns of most genes under osmotic stress and salt stress were consistent, except for *GhPIP1;2_A/D*.

**FIGURE 3:**
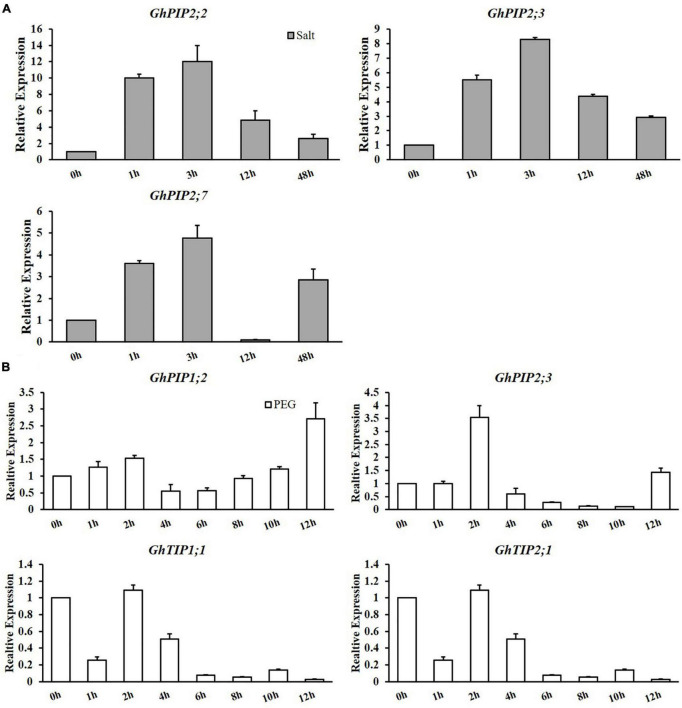
Expression patterns of candidate genes under salt and drought stresses. **(A)** Salt stress (150 mM NaCl) and **(B)** drought stress (15% PEG6000). Quantitative real-time PCR (qRT-PCR) was used to investigate the expression levels of candidate genes. *GhUBQ7* was used as the internal control to calculate and normalize the expression levels. *P < 0.05, **P < 0.01.

To validate the expression pattern of *GhPIP*s under salt and osmotic stresses, we performed qRT-PCR (Figure 3 and Supplementary Figure S2). For easy description, we used *GhPIP* to represent the *GhPIP_A/D* gene pair. Among the 21 gene pairs, most *GhPIP* genes were significantly induced after 150 mM NaCl treatment, while the expression of nine genes (*GhPIP1;6, GhPIP1;11, GhPIP2;1, GhPIP2;11, GhPIP1;2, GhPIP1;4, GhPIP2;8, GhPIP2;10*, and *GhPIP2;13*) was not detectable. A total of ten pairs of *GhPIPs* (*GhPIP1;3, GhPIP1;7, GhPIP1;9, GhPIP1;10, GhPIP2;2, GhPIP2;3, GhPIP2;5, GhPIP2;6, GhPIP2;7*, and *GhPIP2;9*) were upregulated after salt stress. Among ten upregulated *GhPIP* genes, five (*GhPIP1;10, GhPIP2;2, GhPIP2;3, GhPIP2;5*, and *GhPIP2;7*) showed the highest expression at 3 h, which is consistent with the RNA-seq result. Six pairs of *GhPIP* genes (*GhPIP1;1, GhPIP 1;5, GhPIP 1;8, GhPIP 2;4, GhPIP 2;12*, and *GhPIP* 2;14) were alternately up- and downregulated throughout time courses of the treatment; however, these genes showed sharp upregulation at 48 h. Three genes (*GhPIP2;4, GhPIP2;12*, and *GhPIP2;14*) were significantly downregulated at 12 h, while *GhPIP1;8* was downregulated at 3 h. *GhPIP1;1* showed minor downregulation at 1 h and stayed to 12 h but upregulated dramatically at 48 h. *GhPIP1;5* genes remained downregulated from 1 to 3 h, after that, upregulated until 48 h.

We observed that four genes (*GhPIP1;2, GhPIP2;3, GhTIP1;1*, and *GhTIP2;1*) were upregulated at the early stages after drought stress. To verify their expression levels, we performed qRT-PCR (Figure 3B). The expression of these genes reached the peak value at 2 h after osmotic stress except for *GhPIP1;2*, suggesting that these genes functioned in osmotic stress response. All the results showed that *GhPIPs* genes might be involved in the abiotic stress of cotton.

### *Cis*-Regulatory Elements of *GhPIP*s and *GhTIP*s

*Cis*-regulatory sequences are linear non-coding DNA fragments that exist in front of the promoter region. *Cis*-regulatory elements have various functions, which depend on their types, locations, and orientations. To expound the function of *GhPIP*s and *GhTIP*s, 1,500-bp upstream sequences of *GhPIP*s promoter regions were extracted and used to predict *cis*-elements using the PlantCARE database. A total of 272 *cis*-regulatory elements were detected in *GhPIP*s and *GhTIP*s. Notably, 85% were core promotor elements or binding sites of DNA binding protein. In addition, 53, 23, and 10% of the motifs were involved in light response, plant hormone-responsive, and other stress-responsive, yet 14% were undefined. These results demonstrated that *GhPIP*s and *GhTIP*s have multiple roles in cotton developmental processes and abiotic stress response. In this study, we focused on five *cis*-regulatory elements responding to abiotic stresses, including a *cis*-acting regulatory element essential for the anaerobic induction (ARE), a *cis*-acting element involved in low-temperature responsiveness (LTR), an MYB binding site involved in drought inducibility (MBS), a motif involved in differentiation of the palisade mesophyll cells (HD-Zip 1), and a wound-responsive element (WUN-motif) (Supplementary Table S3 and Supplementary Figure S2). In general, *GhPIP* genes possessed at least one stress-response-related *cis*-element. In this study, we discovered that most *GhPIP* genes had an ABA-responsive element (ABRE) that participated in ABR signaling pathways under salt stress (Supplementary Figure S2).

### Silencing of *GhPIP2;7* Decreased Salt Tolerance in Cotton

The *GhChlI*-silenced plants showed a typical photobleaching phenotype in newly grown leaves, which indicated that the VIGS system was applied successfully in GX100-2 (Supplementary Figure S3A). We also examined the expression levels of three genes (*GhPIP2;2, GhPIP2;3*, and *GhPIP2;7*) in gene-silenced plants by qRT-PCR. The gene expression was significantly decreased in gene-silenced plants than that in mock (plants transformed in empty vector) (Supplementary Figure S3B).

**FIGURE 4:**
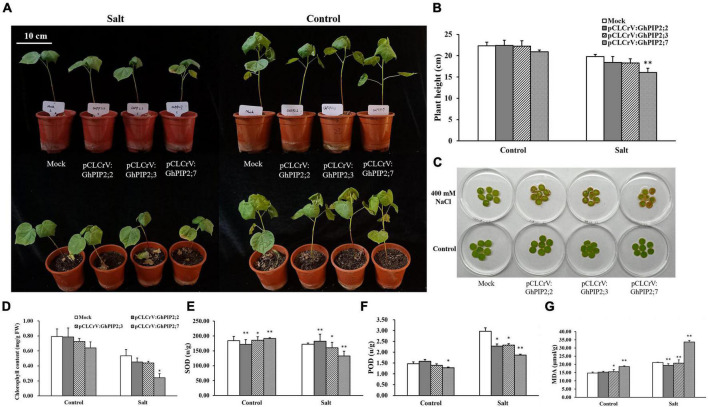
Silencing of *GhPIP2;7* decreased tolerance to salt stress in upland cotton. **(A)** Phenotypes of *GhPIP2;2-*, *GhPIP2;3-*, and *GhPIP2;7-*silenced plants under salt stress and normal growth conditions. Plants inoculated with cotton leaf crumple virus-A (CLCrVA) were used as mock (Bar = 10 cm). **(B)** The plant height of *GhPIP2;2-*, *GhPIP2;3-*, and *GhPIP2;7*-silenced plants under salt stress and normal growth conditions. **(C)** Leaf disks of mock and *GhPIP2;2-*, *GhPIP2;3-*, and *GhPIP2;7*-silenced plants incubated in 400 mM NaCl or deionized water for 4 days. **(D)** Chlorophyll content of mock and *GhPIP2;2-*, *GhPIP2;3-*, and *GhPIP2;7*-silenced plants incubated in 400 mM NaCl or deionized water for 4 days. **(E–G)** Represent the chlorophyll content, malondialdehyde (MDA) concentration, superoxide dismutase (SOD), and peroxidase (POD) activities of leaves in *GhPIPs*-silenced plants under 400 mM NaCl stress, respectively. Data are the mean of three replications ± SE. (*P < 0.05, **P < 0.01, *t*-test, *n* = 3).

To further evaluate the phenotype of the gene-silenced plants under salt stress, the plants were treated with 400 mM NaCl for 12 days. Under salt stress conditions, the leaves of *GhPIP2;7*-silenced plants wilted more seriously, and the plant height decreased than that in mock and other gene-silenced plants (*GhPIP2;2* and *GhPIP2;3*) (Figures 4A,B). Furthermore, the leaf disks of gene-silenced plants were incubated in 0 or 400 mM NaCl solutions for 4 days (Figures 4C,D). The leaf disks of *GhPIP2;2-* and *GhPIP2;3*-silenced plants did not produce any observable differences compared to those of mock under normal conditions. However, the leaf disks of *GhPIP2;7-*silenced plants showed more significant browning than those from the mock (Figure 4C). The chlorophyll content of leaf disks in *GhPIP2;7*-silenced plants decreased significantly, but the changes of *GhPIP2;2-* and *GhPIP2;3-*silenced plants were not significant after salt stress treatment (Figure 4D). Subsequently, we tested the MDA content, POD, and SOD activities in the leaves (Figures 4E–G). Under the control condition, the SOD activity and the MDA contents were increased, while the POD activity decreased significantly in *GhPIP2;7*-silenced plants. Salinity greatly reduced the activity of antioxidant enzymes (SOD and POD) and increased the MDA content in *GhPIP2;7*-silenced plants. The silencing of *GhPIP2;2* and *GhPIP2;3* in cotton did not produce any observable differences in phenotype and plant height, compared to the mock under salt stress conditions (Figures 4A,B). The trends of antioxidant enzyme activity and MDA content were not consistent under salt stress conditions (Figures 4E–G). The results indicated that the silencing of *GhPIP2;7* significantly decreased cotton tolerance to salt stress.

### Silencing of *GhTIP2;1* Decreased Drought Tolerance of Cotton

**FIGURE 5:**
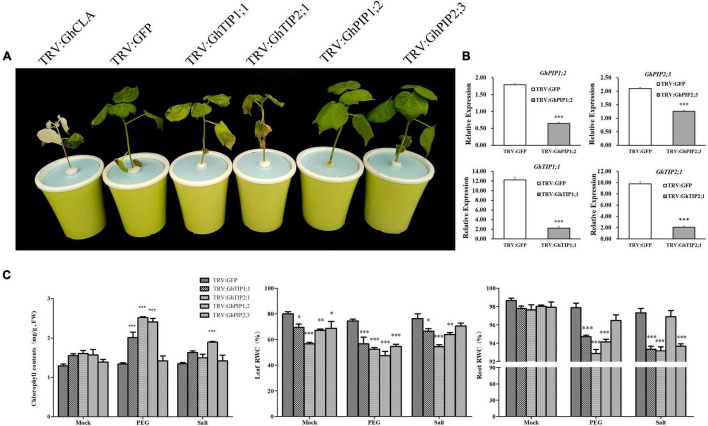
Silencing of *GhTIP1;1* and *GhTIP2;1* decreased tolerance to osmotic stress in cotton plants. **(A)** Phenotypes of *GhTIP1;1*, *GhTIP2;1-*, *GhPIP1;2-*, and *GhPIP2;3-*silenced plants treated with 13% PEG6000 for 3 days. Plants inoculated with tobacco rattle virus (TRV):GFP were used as mock. **(B)** The relative expression levels of candidate genes in gene-silenced plants. **(C)** Chlorophyll content, leaf relative weight content (RWC), and root RWC of mock and gene-silenced plants incubated in 13% PEG6000. Data are the mean of three replications ± SE. (*P < 0.05, **P < 0.01, ***P < 0.001, *t*-test, *n* > 3).

Four genes (*GhTIP1;1, GhTIP2;1, GhPIP2;1*, and *GhPIP2;3*) were selected to validate their functions in drought stress response using the VIGS experiment. We found that the silencing of *GhTIP1;1* and *GhTIP2;1* resulted in the wilting and yellowing of the whole plant under drought stress (Figure 5A). The gene expression was significantly decreased in the gene-silenced plants than that in mock (Figure 5B). The chlorophyll content was increased in TRV:GhTIP2;1 plants after PEG treatment, while the RWC in roots and leaves was reduced dramatically in TRV:GhTIP1;1 and TRV:GhTIP2;1 plants under drought stress (Figure 5C). The results indicated that the silencing of *TIP2;1* decreased the drought tolerance in cotton.

### The Overexpression of *GhTIP2;1* Improves Drought Tolerance of *Arabidopsis*

**FIGURE 6:**
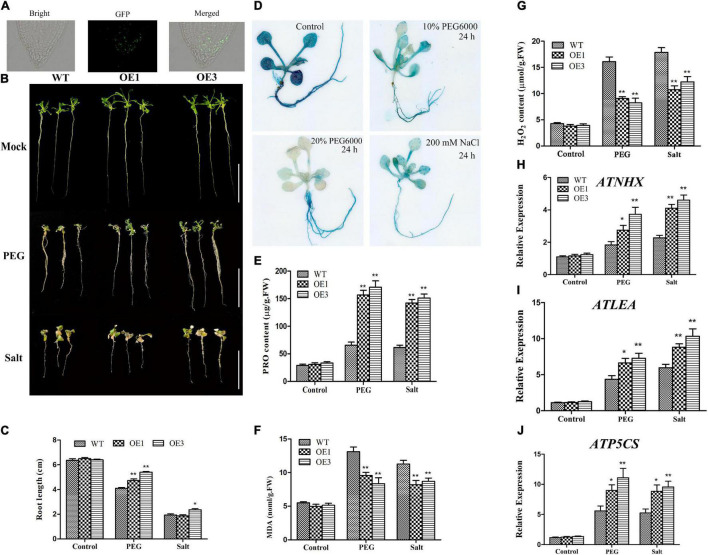
Overexpression (OE) of *GhTIP2;1* enhanced drought tolerance in *Arabidopsis.* **(A)** Subcellular localization of GhTIP2;1 protein in transgenic *Arabidopsis* roots. **(B)** Phenotypes of *GhTIP2;1* overexpressing (OE) *Arabidopsis* treated with 15% PEG6000 and 150 mM NaCl. **(C)** Root length in *GhTIP2-1* overexpressing lines under salt and osmotic stresses (Bar = 2 cm). **(D)** Histochemical glucuronidase (GUS) assays in Pro*GhTIP2;1*:GUS transgenic *Arabidopsis* plants. The 7-day-old seedlings grown in ^1^/_2_ MS medium (control), treated with 10% PEG6000, 20% PEG6000, and 200 mM NaCl for 24 h. The analysis of H_2_O_2_ **(E)**, Proline **(F)**, and MDA **(G)** contents in *GhTIP2-1* overexpressing lines under salt and osmotic stresses (*n* > 3, *P < 0.05; **P < 0.01). Gene expression pattern of stress-related genes in OE lines and wild type (WT) plants *AtNHX* **(H)**, *AtLEA* **(I)**, and *AtP5CS* **(J)**.

To evaluate the function of *GhTIP2;1* in response to salt and osmotic stresses, two independent homozygous lines of the T_3_ generation (OE1 and OE3) were used for the subsequent physiological experiment. The OE vector pCAMBIA1300-GhTIP2;1-eGFP contains a GFP label, so the transgenic *Arabidopsis* lines can be identified by detecting GFP signals. The GFP signals were detected in the root of transgenic *Arabidopsis*, which indicated that *GhTIP2;1* had been transferred into the *Arabidopsis* genome successfully (Figure 6A). The 1-week-old *GhTIP2;1* overexpressed lines (OE1 and OE2) and WT seedlings were transferred to ^1^/_2_ MS medium containing 15% PEG6000 and 150 mM NaCl, and the root length was measured after 1 week. Under control conditions, there were no phenotype differences observed between WT and OE lines. Root growth was inhibited more seriously in WT than that in OE lines under PEG treatment (Figures 6B,C). The expression pattern of *GhTIP2;1* was determined in transgenic plants, harboring the *GhTIP2;1* promoter that could drive the expression of the *GUS* reporter gene. The *GUS* gene was strongly expressed in cotyledons, rosette leaves, and roots in the control (Figure 6D). After 10% PEG6000 treatments and 200 mM NaCl, the GUS signal was weak in the cotyledons and rosette leaves compared with the control seedlings. GUS staining was inconspicuous in the leaves when treated with 20% PEG6000. These results indicated that *GhTIP2;1* was highly expressed in roots but downregulated in leaves after salt stress and osmotic stress.

In addition, we further analyzed the antioxidant enzyme activity in *GhTIP2;1-*OE plants and WT under salt and osmotic stresses (Figures 6E–G). To evaluate the role of *GhTIP2;1* in the oxidative stress pathway, proline, MDA, and H_2_O_2_ contents were measured in *GhTIP2;1-*OE plants and WT. The results showed that less MDA and H_2_O_2_ accumulated in transgenic plants than that in WT under both salt and osmotic stresses. Also, the proline content was significantly higher in transgenic plants compared to WT under salt and osmotic stresses. These results showed that *GhTIP2;1*-OE plant enhanced osmotic tolerance compared to WT. Furthermore, the expression pattern of stress-responsive genes was determined by qRT-PCR. The transcription levels of stress-responsive genes, including *AtNHX, AtLEA*, and *AtP5CS*, showed no significant difference among OE and WT lines under normal conditions. However, the transcription of these genes in WT and OE lines was significantly induced after salt and drought treatments (Figures 6H–J). Although the transcriptional levels of stress-responsive genes were substantially higher in the OE lines than that in WT plants after NaCl treatment, the phenotype between OE and WT lines under salt stress showed no significant difference in OE1 lines. The above mentioned results indicated that overexpressed *GhTIP2;1* in *Arabidopsis* was more tolerant to drought stress than salt stress treatment.

## Discussion

Plant AQPs stand for a large and diverse family of numerous water channel proteins which are necessary for several physiological processes in living organisms (Tyerman et al., 2021). The success of plant genome sequencing has enabled the identification and characterization of AQPs in *Arabidopsis* (Johanson et al., 2001), *S. lycopersicum* (Reuscher et al., 2013), *P. vulgaris* (Ariani and Gepts, 2015), *M. esculenta* (Putpeerawit et al., 2017), *Z. mays* (Chaumont et al., 2001), *O. sativa* (Sakurai et al., 2005), and banana (Hu et al., 2015). These researches provided models for the identification of the *AQP* gene family in cotton species.

The release of three cotton genome data allowed the identification and characterization of the *AQP* gene family. Four genome versions of *G. hirsutum* acc. TM-1 was released from different organizations, and the differences among these genome versions were mainly concentrated on the duplicated genes (Zhang et al., 2015; Hu et al., 2019; Chen et al., 2020). Taking advantage of the transcriptomic data and gene annotation information of NBI_V1.1 in CottonFGD and CottonGen websites, we analyzed these genes and their proteins by extracting and aligning their sequences in *G. hirsutum* acc. TM-1 (NBI_V1.1) (Zhang et al., 2015). In total, 221 putative AQPs were identified in three cotton species. A total of 54, 56, and 111 *AQP* genes were predicted in *G. arboreum*, *G. raimondii*, and *G. hirsutum*, respectively. Of note, 111 full-length AQP-coding sequences were identified in *G. hirsutum*; thereinto, 48, 26, 20, 11, and 6 members belonging to the PIP, TIP, NIP, SIP, and XIP subfamily, respectively. Most members existed as gene pairs in A*t* and D*t* subgenome of *G. hirsutum*, while only a few members existed in one of the subgenomes, such as *GhPIP1;11* (Supplementary Figure S2 in Supplementary File).

In upland cotton, 71 *AQP* genes were identified and classified into five subfamilies, namely, PIP (28), TIP (23), NIP (12), SIP (7), and XIP (1) based on the expressed sequence tag (EST) sequences from previous research (Park et al., 2010). As shown in Supplementary Tables S1, S2 in Supplementary File, 111 putative *AQP* genes were predicted in *G. hirsutum* by HMMER search in our research, while the number was 113 in the previous research. Two *GhAQP* genes (*GhPIP2;4b_Dt* and *GhPIP2;4d_Dt*) in the *Dt* subgenome were specifically presented in the study by Li W. et al. (2019). We found that three *AQP* genes (*GhPIP2;9_A*, *GhNIP5;1_A*, and *GhSIP2;1_A*) were specifically presented in *G. hirsutum*, and two *SIP* genes were specifically found in *G. arboreum* in our study, while one (*GaNIP7;1b*) and two *AQP* genes (*GrPIP2;7d* and *GrPIP2;8*) were predicted specifically in A_2_ and D_5_ genome, respectively, which is consistent with the results suggested by Li W. et al. (2019). It was found that two *AQP* genes (*GhPIP1;1* and *GhTIP2;1*) were downregulated under salt stress (Braz et al., 2019). Three *PIP* genes (*GhPIP1;1*, *GhPIP2;1*, and *GhPIP2;2*) were isolated from the cotton root cDNA library, and the transcriptional changes of these genes were observed under abiotic stresses (Li et al., 2009). However, to the best of our knowledge, studies focused on the roles of AQP in response to abiotic stress in upland cotton were limited.

### The Expansion and Duplication of Aquaporins in *Gossypium*

In this study, we analyzed the AQPs of *G. hirsutum, G. raimondii, G. arboreum*, and other 34 plant species and found that the number of AQPs was consistent with the total gene number of eudicots, not monocots (Supplementary Figure S1 in Supplementary File). Before the formation of angiosperms, all plant genomes experienced two whole-genome replication events, in which eudicots and monocots experienced genome tripling and replication events, respectively (Wu et al., 2020).

Upland cotton, which is a natural allopolyploid, is an excellent plant material to explore the mechanism of genome evolution and polyploidy formation. Gene duplication is an important mechanism for increasing genetic variability and creating novel genes in plants (Moore and Purugganan, 2003). Previous analyses on biotin carboxyl carrier protein (BCCP) and phospholipase C (PLC) gene evolution in *Gossypium* revealed that the duplicated genes evolved independently after polyploidy formation (Cui et al., 2017; Zhang et al., 2018). To further understand the duplication events, we investigated the expansion mechanism of *GhAQP* genes. A total of 115 duplicated gene pairs were identified, and most of those were distributed on different chromosomes (Supplementary Figures S5, S6 and Supplementary Table S3 in Supplementary File). The result demonstrated that the expansion of *GhAQP* genes was mainly caused by segmental duplication. The number of AQP-coding genes in *G. hirsutum* was approximately the sum of *G. raimondii* and *G. arboreum*, according to the WGD event in cotton evolution (Supplementary Figure S5 and Supplementary Table S3 in Supplementary File). In this study, we observed that *AQP* genes in the A genome and A*t* subgenome had common ancestors, as well as in the D genome and D*t* subgenome, which indicated that *AQP* genes were highly conserved during cotton evolution. During the long history of plant evolution, genes have been exposed to different selective pressures, including positive selection, negative selection, and purifying selection (Flagel and Wendel, 2009). The average *Ka/Ks* ratio of 115 *GhAQP* gene pairs was less than 1, which indicated that *GhAQP* genes experienced purifying selection during evolution (Supplementary Table S3 in Supplementary File).

### Conservation and Differentiation of Aquaporins in *Gossypium*

The evolutionary analysis on *Gossypium AQP* genes showed that most of them were greatly conserved during evolution. AQPs have typical conserved NPA motifs and ar/R selectivity filter features, which are indispensable in determining the transport channel specificity (Maurel et al., 2015). All the members of the PIP, NIP, and SIP subfamily and most of the TIPs contained the same ar/R selectivity filter. PIPs showed typical NPA motifs and highly conserved ar/R selectivity filter (F-H-T-R), which are the typical water-transporting configuration (Supplementary Table S1 in Supplementary File). These two motifs were highly conserved in PIPs of *A. thaliana* (Johanson et al., 2001), *Z. mays* (Chaumont et al., 2001), *S. lycopersicum* (Reuscher et al., 2013), and *Brassica rapa* (Kayum et al., 2017). TIPs exhibited four different forms of ar/R selectivity filter, namely, GhTIP1;1 (H-I-D-V), GhTIP2;2 (H-I-S-R), GhTIP3;1 (H-I-D-R), and GhTIP5;1 (N-V-S-L), which provided evidence for the variability of TIP subfamily (Sun et al., 2016). The NIP subfamily was quite divergent in NPA motifs and ar/R selectivity filter compared to other subfamilies in upland cotton, suggesting that the substrates for transport were diverse (Perez et al., 2014). This finding suggested that the domain of the PIP subfamily was more conserved than other subfamilies. Most PIPs, SIPs, and XIPs were predicted to be positioned on the plasma membrane, suggesting that they may regulate osmotic potential and water flows across this essential plant subcellular compartment (Figure 1 and Supplementary Table S1 in Supplementary File). TIPs were mainly located on the vacuole membrane, suggesting that the TIPs may regulate cellular osmosis and water homeostasis in cotton (Figure 1 and Supplementary Table S1 in Supplementary File). The ar/R filter in the members of different subfamilies was quite divergent, indicating their divergence in solute permeability (Supplementary Table S1 in Supplementary File).

Salt and drought stresses are the major abiotic threats to plants that affect plant growth and reduce crop yield. Excess salt may become cytotoxic to the plant, leading to cell membrane destruction (Zhu, 2001). Most homologous *AQP* genes in A*t* and D*t* subgenomes showed the same expression pattern under salt or drought stress (Figure 2). The *PIP* genes played an important role in conferring abiotic stress tolerance in plants, including drought, cold, and salt (Lu et al., 2018; Wang et al., 2019). Most of the *GhPIP* genes or gene pairs were rapidly induced when exposed to salt stress and osmotic stress, except for *GhPIP1;2_A* and *GhPIP1;2_A/D* (Figure 2). The structures of the PIP subfamily were highly conserved, which may explain the similar biological functions in response to abiotic stress (Supplementary Figure S1 and Supplementary Table S1 in Supplementary File). It demonstrated that most PIPs are conserved in response to abiotic stress, but the functions of a few genes are differentiated.

A total of ten *GhPIP* genes (*GhPIP1;3, GhPIP1;7, GhPIP1;9, GhPIP1;10, GhPIP2;2, GhPIP2;3, GhPIP2;5, GhPIP2;6, GhPIP2;7*, and *GhPIP2;9*) were significantly induced (Log_2_-based value > 1) after 150 mM NaCl treatment, suggesting that *GhPIP* genes response to salt stress extensively (Figure 3 and Supplementary Figure S1). Most *GhPIP* genes showed similar expression patterns between A*t* and D*t* subgenome (Supplementary Table S4; Li W. et al., 2019). In our study, the expression of most *GhPIPs* was increasing at early stages after salt stress, for instance, *GhPIP1;10*, *GhPIP2;5*, and *GhPIP2;7* showed the high expression at 3 h after salt stress, *GhPIP1;8* reached the high level at 1 h, and *GhPIP2;9* reached the peak at 12 h (Figure 3 and Supplementary Figure S1). The expression of *GhPIPs* increased rapidly under salt stress from 12 to 24 h and reached a high expression level at 24 h except for *GhPIP1;4a*_A*t* (D*t*), which reached its expression peak at 48 h, which explained the mechanism of leaves wilting in a short period after salt stress (Li W. et al., 2019). The expression of *GhPIP2;7* reached a high level at 3 h after salt stress and then continuously downregulated to 48 h in this study using *G. hirsutum* GX100-2 (leaves); the expression of *GhPIP2;1* (*GhPIP2;7* in our research) increased rapidly under salt stress from 12 to 24 h and reached a high level at 24 h in *G. hirsutum* acc. TM-1 (root) (Figure 3; Li W. et al., 2019). Our data indicated that the response speed to salt stress was strongly related to the tissues or varieties in upland cotton.

### Silencing of *GhPIP2;7* Decreased the Salt Tolerance in Upland Cotton

Previous reports had demonstrated that the *PIP* genes played a vital role in response to salt stress and could actively regulate root and leaf hydraulics in plants (Li G. et al., 2014). *GhPIP2;2, GhPIP2;3*, and *GhPIP2;7* were significantly upregulated in response to salt stress, which was consistent with the results in *Beta vulgaris* (Skorupa-Kłaput et al., 2015). *AtPIP2;4* and *AtPIP2;5* exhibited the upregulated expression under salt stress (Feng et al., 2018). *PIP2;7*, which was initially referred to as Salt-Induced *MIP* (*SIMIP*), was reported to be strongly upregulated by 150 mM NaCl treatment in the 2-week-old *Arabidopsis* seedlings (Jang et al., 2004). The OE of *PePIP2;7* enhanced salt and drought stress tolerance of *Arabidopsis* and yeast (Sun et al., 2021). *MsPIP2;2* conferred salt tolerance by regulating antioxidant defense system-mediated reactive oxygen species (ROS) scavenging, K^+^/Na^+^ homeostasis, and stress-responsive gene expression in *Arabidopsis* (Li S. et al., 2019). Taken together, salt stress stimuli resulted in a wide variety of *PIP* gene expression patterns. *GhPIP2;2* and *GhPIP2;3* were the homologous genes of *AtPIP2;4*, which showed the same expression pattern after 150 mM NaCl treatment (Figure 3 and Supplementary Figure S2 in Supplementary File). Plants showed no obvious salt damage phenotype whether *GhPIP2;2* or *GhPIP2;3* were silenced (Figure 4), which may be due to the functional redundancy of homologous genes.

There was no difference in phenotype between *GhPIP*s-silenced plants and control under normal conditions (Figure 4). The salt injury symptoms of *GhPIP2;7*-silenced plants were more severe than that in mock plants, which included yellowing, slight wilting, and dwarfing (Figures 4A,B). MDA is the product of the peroxidation reaction, which indicates the degree of peroxidation of the cell membrane and the strength of the stress reaction. Antioxidant enzymes can alleviate oxidative damage caused by salt stress in plants. Under salt stress conditions, the activity of antioxidant enzymes (SOD and POD) in *GhPIP2;7*-silenced plants decreased dramatically, while the MDA content increased significantly, which aggravated the salt injury phenotype of plants (Figures 4E–G). We found that the HD-Zip 1 element, which was involved in the differentiation of palisade mesophyll cells, existed in *GhPIP1;9_A/D* and *GhPIP2;7_A/D*. It was reported that PIP2;1 contributed to ABA-triggering stomatal closure through open stomata (OST)1-mediated phosphorylation (Grondin et al., 2015). Combined with the *cis*-elements in promoter and expression profile of *GhAQPs* under salt stress, *GhPIP2;7* may play a positive regulatory role in response to salt stress, which affects water transport by controlling mesophyll expansion. However, the roles of PIPs in response to salt tolerance in cotton still need further research.

### *GhTIP2;1* Increases Tolerance to Osmotic Stress by Accumulating More Proline and Improving the Na^+^ Efflux

The silencing of *GhTIP1;1* and *GhTIP2;1* resulted in the wilting and yellowing of the whole plant under drought stress (Figure 5A). The RWC in root and leaves was reduced in TRV:GhTIP1;1 and TRV:GhTIP2;1 plants under drought stress (Figure 5C). *GhTIP2;1* may be the key gene involved in drought stress response. To further validate its roles, we overexpressed it in *A. thaliana* under osmotic stress. In this study, the expression pattern of *GhTIP2;1* was determined by analyzing transgenic plants harboring the *GhTIP2;1* promoter that could drive the expression of the *GUS* reporter gene. *GhTIP2;1-*OE individuals grow significantly better than WT in *Arabidopsis* under drought stress (Figure 6A).

Proline is an osmolyte that plays an important role in oxidative stress response. The accumulation of H_2_O_2_ in the plant cell could cause oxidative damage, while its lower concentration correlated with drought tolerance (Ullah et al., 2018). The MDA level under stress conditions was an indicator of ROS destructive effects (Sharma et al., 2012). More proline accumulation and less MDA and H_2_O_2_ contents in transgenic plants suggested that the OE of *GhTIP2;1* reduced the sensitivity of *Arabidopsis* to drought stress. NHX is Na^+^/H^+^ antiporters that maintain cellular Na^+^/K^+^ and pH homeostasis (Long et al., 2020). The upregulation of *ATP5CS* mainly promoted the accumulation of proline (Trovato et al., 2018). To investigate the role of *GhTIP2;1* in osmotic stress, we analyzed the expression of three stress-related genes (*AtP5CS, AtNHX*, and *AtLEA*). The results showed that *AtP5CS, AtNHX*, and *AtLEA* genes were upregulated in *GhTIP2;1-*OE *Arabidopsis* plants under osmotic stress. Therefore, *GhTIP2;1* may enhance the osmotic tolerance by accumulating more proline and increasing the Na^+^ efflux.

## Conclusion

In this study, a total of 111, 54, and 56 *AQP* genes were identified in three cotton species (*G. hirsutum, G. arboreum*, and *G. raimondii*, respectively). Their conserved motifs and gene structure within the same subfamilies shared a notable similarity, which leads to conserved functions. Some *GhPIP*s and *GhTIP*s were induced significantly in both drought and salt stresses. The silencing of *GhPIP2;7* severely compromised the salt tolerance of upland cotton, while *GhTIP2;1* acted as a positive regulator in both transgenic *Arabidopsis* and cotton under drought stress. Our study revealed that *GhPIP2;7* and *GhTIP2;1* positively regulated the tolerance of upland cotton under salt and osmotic stresses, respectively, and these two *AQP* genes provide new resources for the genetic improvement of salt and drought tolerance in upland cotton.

## Data Availability Statement

The original contributions presented in the study are included in the article/Supplementary Material, further inquiries can be directed to the corresponding author/s.

## Author Contributions

AG and JFH performed bench experiments, data analysis, and manuscript preparation. BL and MZ participated in VIGS experiments. YS, NZ, and YH attended the discussion. JPH, GS, and BT designed the experiments and provided a research platform. JPH, NZ, and YH revised the manuscript. All authors approved the final manuscript.

## Conflict of Interest

The authors declare that the research was conducted in the absence of any commercial or financial relationships that could be construed as a potential conflict of interest.

## Publisher’s Note

All claims expressed in this article are solely those of the authors and do not necessarily represent those of their affiliated organizations, or those of the publisher, the editors and the reviewers. Any product that may be evaluated in this article, or claim that may be made by its manufacturer, is not guaranteed or endorsed by the publisher.

## Footnotes

1
https://wolfpsort.hgc.jp/

## Supplementary Material

Supplementary File Conservation and differentiation of AQPs in *Gossypium.*

Supplementary Figure S1 Expression patterns of candidate genes under salt stress. The expression patterns of candidate genes under salt stress (150 mM). Quantitative RT-PCR was used to investigate the expression levels of candidate genes. *GhUBQ7* was used as the internal control to calculate and normalize the expression levels.

Supplementary Figure S2 Predicted *cis*-elements respond to abiotic stress in promoter regions of *GhAQP*s. ARE, a *cis*-acting regulatory element essential for the anaerobic induction; LTR, a *cis*-acting element involved in low-temperature responsiveness; MBS, MYB-binding site involved in drought inducibility; HD-Zip 1, motif involved in the differentiation of the palisade mesophyll cells, WUN-motif, wound-responsive element.

Supplementary Figure S3 Silencing efficiency and phenotype identification of *GhPIP2;2-*, *GhPIP2;3-*, *GhPIP2;7-*, and *GhChlI*-silenced plants in cotton. **(A)** The phenotype of cotton plants infiltrated with the CLCrV-*ChlI*-vector (positive control) after 14 days. **(B)** The relative expression level of the target gene in cotton infiltrated with the CLCrV-based empty vector (mock), CLCrV- *ChlI* (positive control), CLCrV-*GhPIP2;2*, CLCrV-*GhPIP2;3*, and CLCrV- *GhPIP2;7* vectors. **Represents that the expression of the gene was significantly different from that of the control. Data are the mean of three replications ± SE.

Supplementary Table S1 Primer sequences of candidate genes used in qRT-PCR.

Supplementary Table S2 Primer sequences of candidate genes in vector construction in VIGS experiment and subcellular localization.

Supplementary Table S3 Identification of putative *cis*-regulatory elements in the promoters of *GhPIP*s and *GhTIP*s.

Supplementary Table S4 Comparison of aquaporin gene names between published research and present research.

## Abbreviations

AQP: aquaporin
CLCrV: cotton leaf crumple virus
GUS: glucuronidase
kDa: kilodalton
MDA: malondialdehyde
MW: molecular weight
NIP: nodulin 26-like intrinsic protein
NPA: Asn-Pro-Ala
pI: isoelectric point
PCR: polymerase chain reaction
PIP: plasma intrinsic protein
POD: peroxidase
RWC: relative water content
SIP: small basic intrinsic protein
SOD: superoxide dismutase
TIP: tonoplast intrinsic protein
TMD: transmembrane domains
VIGS: virus-induced gene silencing
XIP: uncategorized X intrinsic protein
qRT-PCR: quantitative real-time PCR.
