# Supplementary material for: Two Aquaporin Genes, GhPIP2;7 and GhTIP2;1, Positively Regulate the Tolerance of Upland Cotton to Salt and Osmotic Stresses
Source: Front Plant Sci. 2022 Feb 11;12:780486. doi: 10.3389/fpls.2021.780486 (PMC8873789; doi:10.3389/fpls.2021.780486)
Supplement: Supplementary File — Conservation and differentiation of AQPs in Gossypium. [file Data_Sheet_1.doc]

**Supplementary File**

**Identification and phylogenetic analysis of *AQP* genes in three *Gossypium* species**

A total of 54, 56, and 111 candidate AQP genes were predicted in *G. arboreum* (A_2_), *G. raimondii* (D_5_)*, and* *G. hirsutum* (AD_1_)*,* respectively (Supplementary Table 1)*.* The number of *AQPs* in *G. hirsutum* was larger than that in *G. arboreum* and *G. raimondii*. That may because upland cotton was derived from the hybridization of two diploid progenitors with doubled chromosomes. The nomenclature of AQPs was based on phylogenetic analysis and sequence similarity. Though the genome size of *G. raimondii* is smaller than that of *G. arboreum,* the number of AQPs in these two diploid cotton species is close.

To study the origin and evolution of cotton AQP genes, the distribution of AQPs in 34 plant species was further analyzed (Supplementary Figure 1A-C). The XIP subfamily was absent in monocots but widely present in dicotyledons except for *Brassica* (Supplementary Figure 1B). The amount of AQPs was the least in *Physcomitrella Patens* (22) and *Selaginella moellendorffii* (18) (Supplementary Figure 1C). The number of AQPs was greater in monocots and eudicots that have experienced genome-wide polyploidization, such as *Zea mays, Gossypium, Brassica rapa, Brassica oleracea, Glycine max, Populus trichocarpa, Populus trichocarpa,* and *Solanum lycopersicum* (Supplementary Figure 1C). In the allotetraploid species, *G. hirsutum* and *Brassica napus*, the number of AQPs is almost equal to the sum of that in two diploid ancestor species. The number of AQPs had no significant correlation with genome size (Supplementary Figure 1D), yet was positively correlated with the total number of genes in eudicots (Supplementary Figure 1E).

Then we explored the evolutionary relationship of AQP genes in *G. raimondii, G. arboreum, G. hirsutum*, *A. thaliana*, *Oryza sativa,* and *Phaseolus vulgaris* (Supplementary Figure 2). No AQP genes of *Arabidopsis* and *O. sativa* clustered in the clade of XIPs. Similar to that in *P. vulgaris*, namely, AQPs in cotton clustered into five distinct subfamilies including PIPs, TIPs, NIPs, SIPs, and XIPs. Compared with the other three species, AQPs from three cotton species had a higher relative coefficient, suggesting a closer relationship (Supplementary Figure 2, Supplementary Figure 3A). The aquaporins of *G. hirsutum* were classified into five subfamilies, that is, 48 PIPs, 26 TIPs, 20 NIPs, 11 SIPs, and 6 XIPs (Supplementary Figure 2). The PIP subfamily (48 members) was divided into PIP1 (20 members) and PIP2 (28 members). The TIP subfamily fell into five groups (TIP1 to TIP5), with 14, 6, 1, 3 and 2 members, respectively. The NIP subfamily branched out into seven groups (NIP1 to NIP7), with 6, 2, 2, 2, 2, 2, and 4 members, respectively. The SIP subfamily split up into SIP1 (nine members) and SIP2 (one member). XIP group had no other branch, with six members. Three *AQP*s (*GhPIP2;9_A, GhNIP5;1_A, GhSIP2;1_A*) and two (*GaSIP1;4, GaSIP1;6*) were specific to *G. hirsutum* and *G. arboreum*, respectively (Supplementary Figure 2; Supplementary Table 2). The result suggests that *AQP*s are usually conserved in *Gossypium*.

**Gene structure and motif analysis of *AQPs* in *Gossypium***

To investigate the roles of aquaporins in upland cotton, ten motifs of 111 GhAQP proteins were screened (Supplementary Figure 3B, Supplementary Figure 4). Eight, four, six, six and six of ten motifs were conserved in PIP, SIP, TIP, NIP, and XIP subfamilies, respectively. Thereinto, motif 1, 4, 5, and 6 were present in all subfamilies; motif 2, 3, 7, and 10 existed specifically in the PIP subfamily; motif 9 is unique to the TIP subfamily; motif 8 occurred in TIP and NIP subfamily. Moreover, three GhPIPs (GhPIP1;1_A/D GhPIP1;2_A/D, GhPIP1;3_A/D) lacked motif 7; GhPIP2;13_A/D and GhPIP2;6_D were deficient of motif 8 and motif 3, respectively. In *Gossypium*, the structure and properties of GhAQPs were conserved in each subfamily, yet vary among subfamilies.

Then, the influence of evolution on the exon-intron structures of *GhAQPs* were studied (Supplementary Figure 3C). Most GhPIP subfamily members had four exons, except for two members (*GhPIP2;6_D*, *GhPIP1;2_A)* that had five and ten exons, respectively. In the TIP subfamily, most members contained three exons, except that *GhTIP4;1_D* and *GhTIP2;3_D* had four exons. In the NIP subfamily, most have five exons except for *GhNIP5;1_A*, *GhNIP6;1_A*, *GhNIP7;1_A*, and *GhNIP7;1_D*. In the SIP subfamily, most had three exons, but *GhSIP1;2_D* had 12 exons, and *GhSIP1;2_A*, *GhSIP1;3_A*, *GhSIP1;4_D*, *GhSIP1;5_A*, and *GhSIP1;5_D* had only one exon. In the XIP subfamily, most of the members had three exons, but *GhXIP2;1_D* just had one exon.

**Chromosomal location and duplication of AQPs in *G. hirsutum***

*AQP* genes were unevenly distributed on the chromosomes, with the number of *AQP* genes on each chromosome varying from one to nine (Supplementary Figure 5). In *G. arboreum*, AQP genes were located in 12 chromosomes and one scaffold, except for chromosome A12. Chromosome A10 contained the largest number (eight) of *GaAQPs* genes. In *G. raimondii*, AQP genes were present on all 13 chromosomes, and the maximum number (nine) of *GrAQP* genes were positioned on chromosome D11. *GhAQP* genes were distributed randomly on 25 chromosomes and nine unmapped scaffolds. Both chromosomes A10 and D10 contained the maximum number (eight) of AQP genes in *G. hirsutum* (Supplementary Figure 5). There were 48 AQPs on 12 A*t* chromosomes, 54 AQPs on 13 D*t* chromosomes, and nine on each of the scaffolds. The number and location of *GhAQPs* in A*t* sub-genome were similar to that in D*t* sub-genome (Supplementary Table 1). The majority of *AQP* genes were located on the distal ends of the chromosomes. The physical locations of the *AQP* genes exhibited great diversity and complexity in the genome of *Gossypium*.

Genome duplication events existed during plant evolution, which may result in the expansion of gene families. However, in *G. hirsutum*, the numbers and locations of duplicated genes were highly symmetric (Supplementary Figure 5, Supplementary Table 3). 115 duplicated gene pairs of AQPs (identity>90%) were recognized in *Gossypium*. Among them, 10, 13, and 92 duplicated gene pairs also existed in the genome of *G. arboreum, G. raimondii,* and *G. hirsutum,* respectively.

In *G. arboreum, G. raimondii,* and *G. hirsutum,* 9, 9, and 84 gene pairs were segmentally duplicated genes, suggesting that segmental duplication played a crucial role in the expansion of the AQP gene family in *G. hirsutum* (Supplementary Figure 6, Supplementary Table 3). Duplicated genes might undergo pseudogenization, sub-functionalization, or neo-functionalization during evolution (Cui et al., 2017). The *Ka/Ks* values of 115 duplicated *GhAQP* pairs were less than 1 (Supplementary Table 3), suggesting that AQPs genes have undergone strong purifying selection pressure after segmental duplication and whole genome duplication (WGD).

**REFERENCE**

Bailey, T.L., Boden, M., Buske, F.A., Frith, M., Grant, C.E., and Clementi, L., et al. (2009). MEME SUITE: Tools for motif discovery and searching. *Nucleic Acids Res.* 37, W202-W208. doi:10.1093/nar/gkp335

Cui, Y., Zhao, Y., Wang, Y., Liu, Z., Ijaz, B., and Huang, Y., et al. (2017). Genome-wide identification and expression analysis of the biotin carboxyl carrier subunits of heteromeric acetyl-coa carboxylase in *Gossypium*. *Front. Plant Sci.* 8, 624. doi:10.3389/fpls.2017.00624

Finn, R.D., Clements, J., and Eddy, S.R. (2011). HMMER web server: Interactive sequence similarity searching. *Nucleic Acids Res.* 39, W29-W37. doi:10.1093/nar/gkr367

Vision, T.J., Brown, D.G., and Tanksley, S.D. (2000). The origins of genomic duplications in *Arabidopsis*. *Science* 290, 2114-2117. doi:10.1126/science.290.5499.2114

Yu, J., Jung, S., Cheng, C., Ficklin, S.P., Lee, T., and Zheng, P., et al. (2014). CottonGen: A genomics, genetics and breeding database for cotton research. *Nucleic Acids Res.* 42, 1229-1236. doi:10.1093/nar/gkt1064

**Additional files**


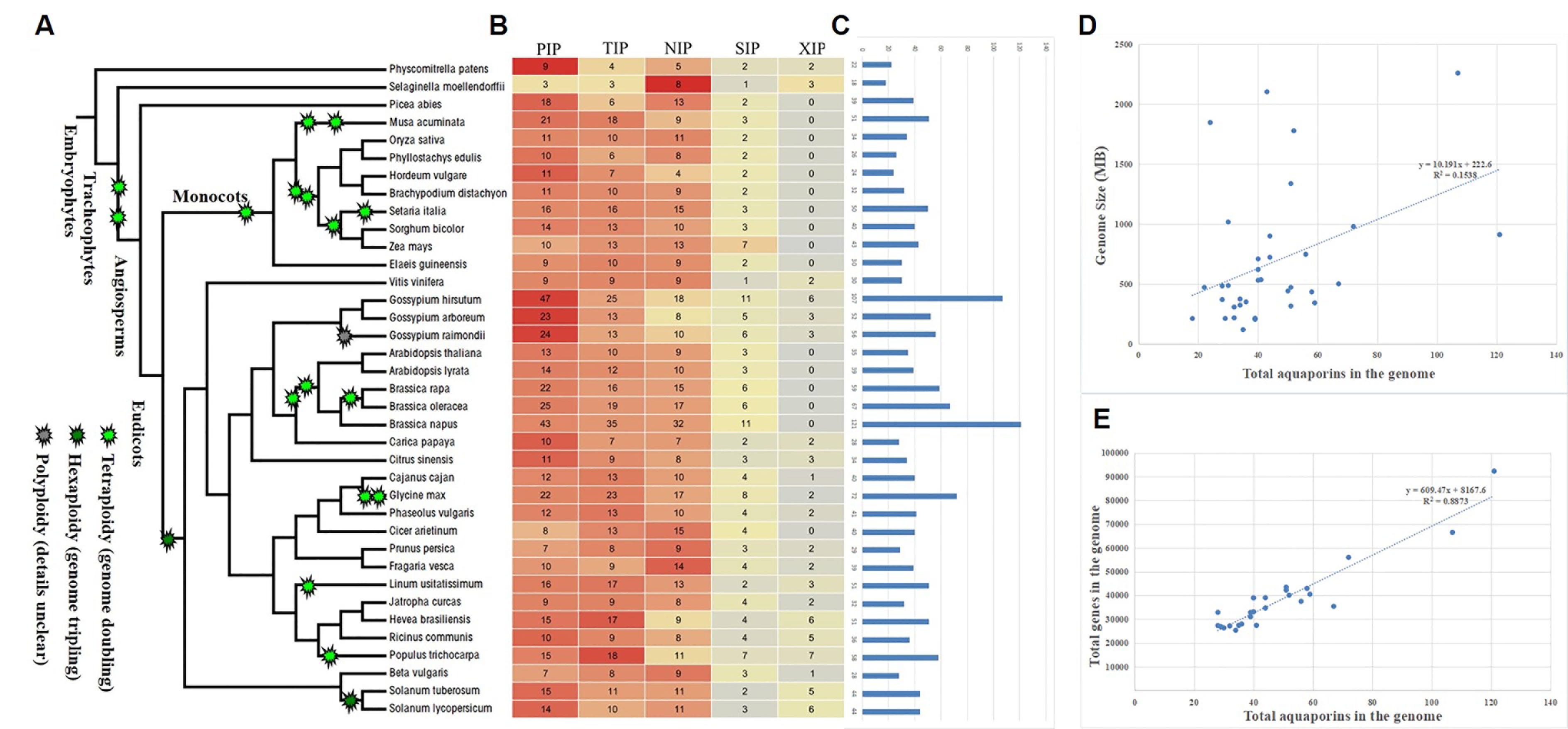


**SUPPLEMENTARY FIGURE S1 |** Plant AQPs and species evolution. **(A)** A phylogenetic tree of 37 identified species of AQPs and genome replication events that accompany the evolution process. **(B)** AQPs family distribution in 37 species. **(C)** Number of AQPs identified in each species. **(D)** The genome size of 37 embryophytes. **(E)** Total genes in the genome of 25 eudicots was plotted against total AQPs identified. Relationship between number of aquaporins (AQPs), genome size and total number of predicted genes in the genome.


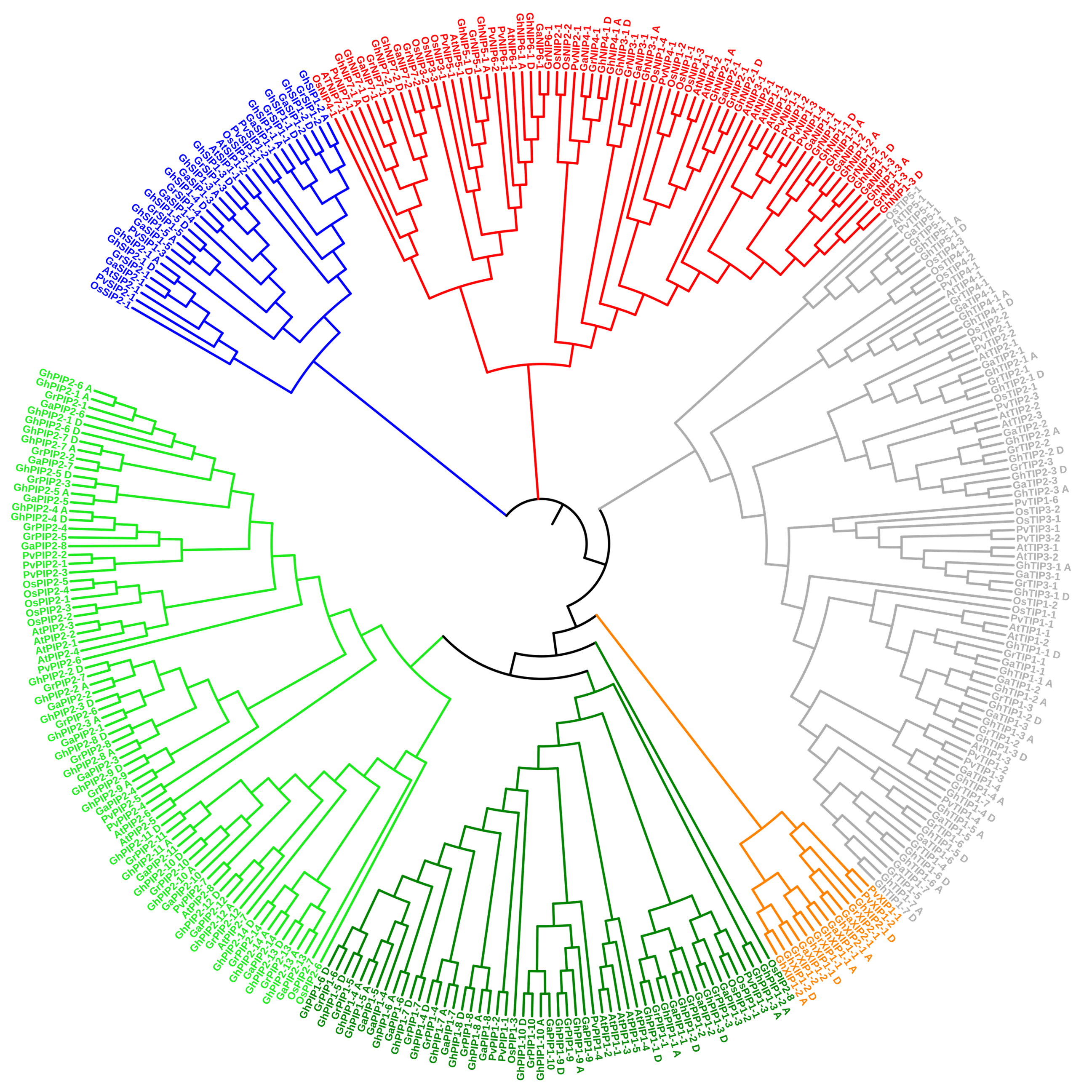
 **SUPPLEMENTARY FIGURE 2 |** Phylogenetic analysis of aquaporins identified in three cotton species and *Arabidopsis thaliana.* The phylogenetic tree was constructed using maximum likelihood (ML) method with 1000 bootstrap replicates. Based on relatedness to characterized proteins, aquaporins were classified as *PIPs* (plasma membrane intrinsic proteins) in the green tree; *TIPs* (tonoplast intrinsic proteins) in grey tree; *NIPs* (nodulin 26-like intrinsic proteins) in the red tree; *SIPs* (small basic intrinsic proteins) in the blue tree, and *XIP*s (X intrinsic proteins) in the orange tree. Ga, Gr, Gh, At, Os and Pv denoted *Gossypium arboreum*, *G. raimondii, G. hirsutum*, *Arabidopsis thaliana*, *Oryza sativa* and *Phaseolus vulgaris*, respectively.


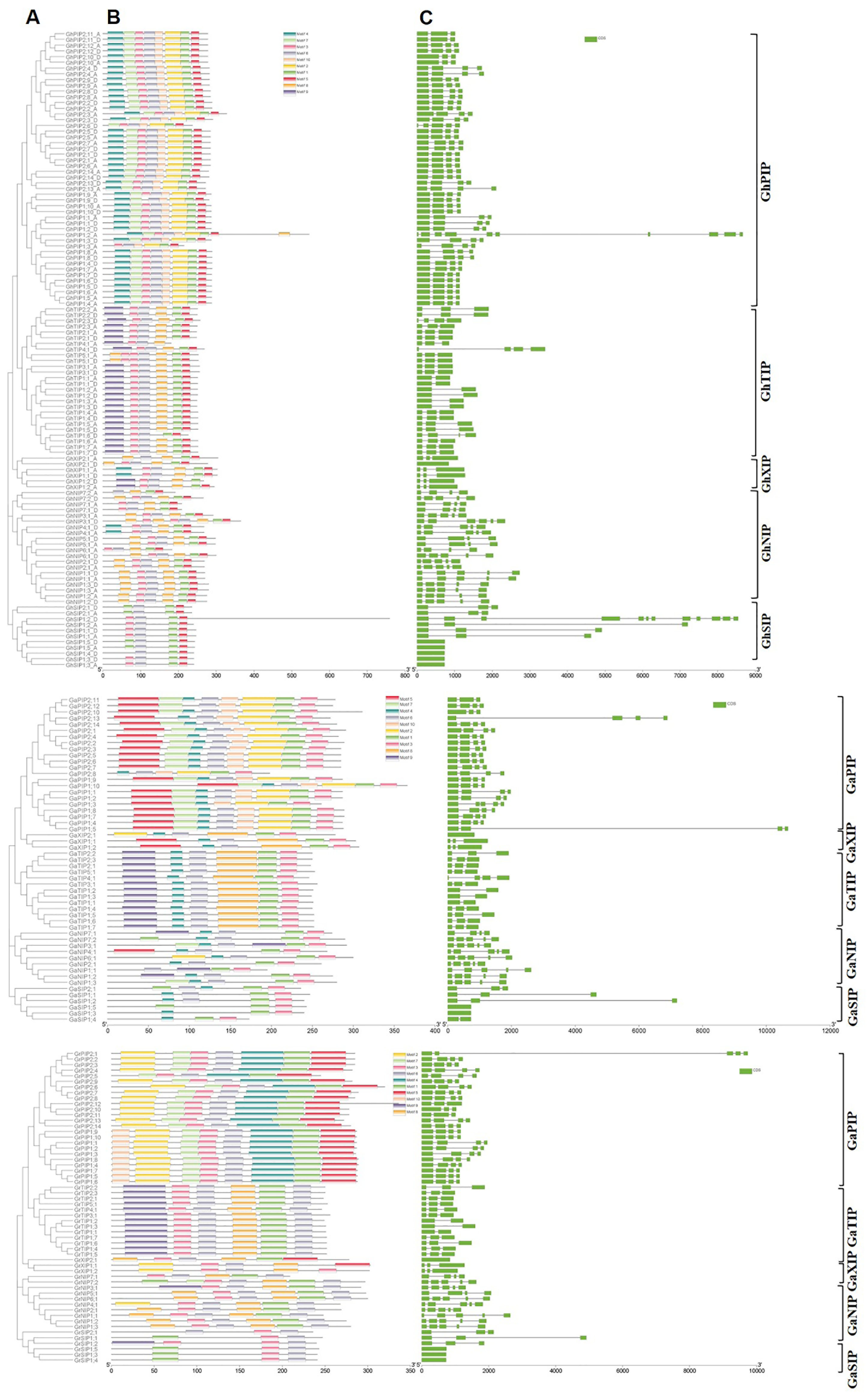
**SUPPLEMENTARY FIGURE 3 |** Phylogenetic relationship, gene structure and motifs of *AQPs* in *Gossypium*. **(A)** Phylogenetic analysis of *AQP* genes in *Gossypium*. **(B)** Motifs of AQPs. **c** Gene structure of *AQP* genes in *Gossypium*. The phylogenetic tree was conducted using MEGA 7.0 software with the maximum likelihood method. Different motifs were shown by different colors and numbered from 1 to 10 in the left. Green boxes represent exons and black lines represent introns in the right. The sizes of exons can be estimated by the scale at bottom.


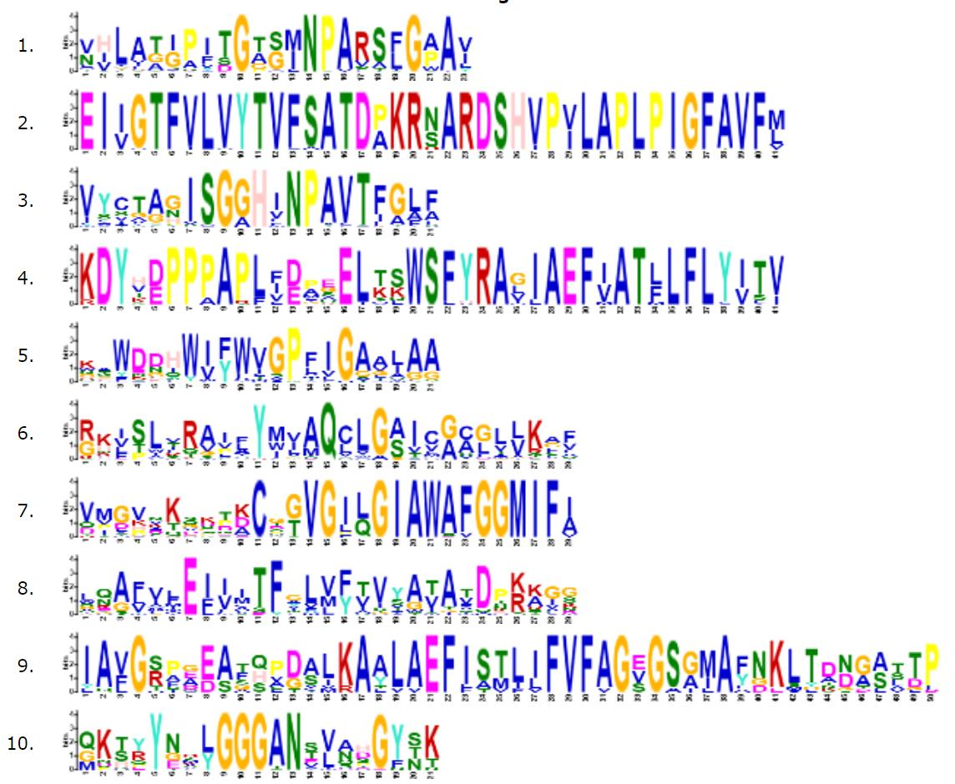


**SUPPLEMENTARY FIGURE 4 |** Sequence information of ten motifs identified by MEME. Different motifs were numbered as 1 to 10.


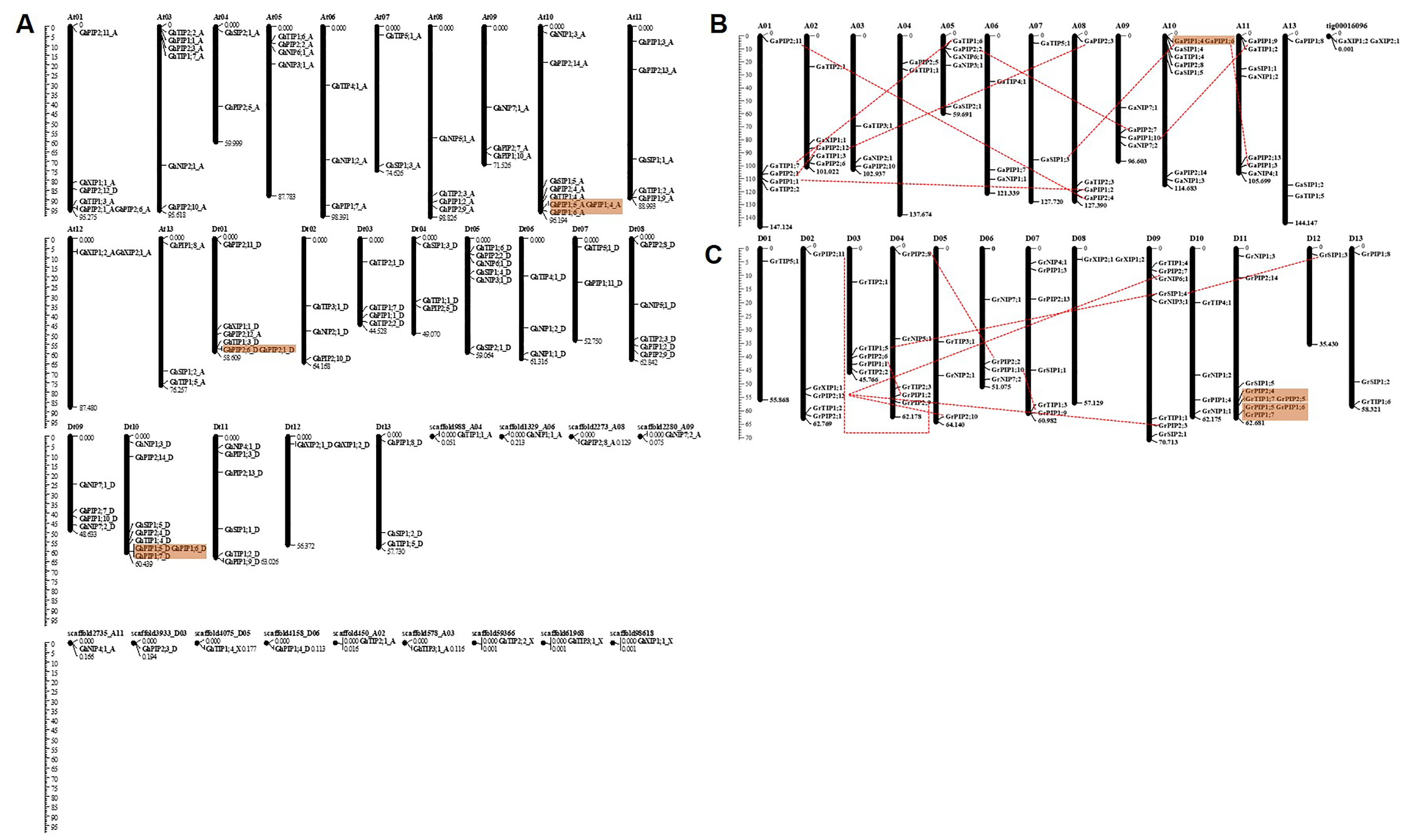


**SUPPLEMENTARY FIGURE 5 |** Chromosomal locations of *AQPs* in *Gossypium.* AQP genes were mapped on different chromosomes in *G. hirsutum* (**A**), *G. arboreum* (**B**), and *G. raimondii* (**C**). The scale bar on the left indicated the length (Mb) of *Gossypium*. The tandem duplicated genes are marked by orange rectangles and the segmentally duplicated genes of *G. arboreum* and *G. raimondii* are connected by red lines between the two relevant chromosomes. The segmentally duplicated genes of *Gossypium* were showed in Supplementary Figure 6.


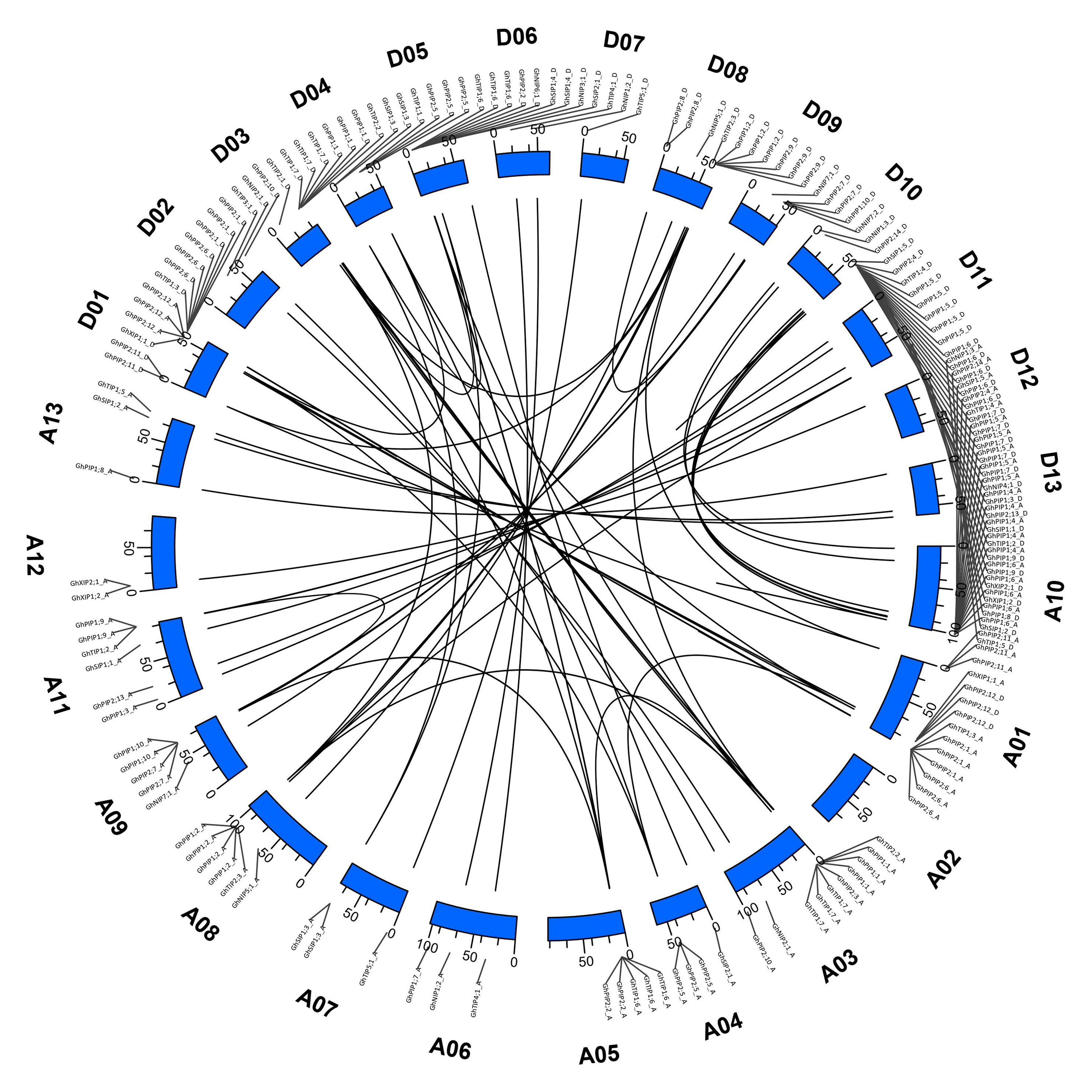


**SUPPLEMENTARY FIGURE 6 |** Chromosomal locations of *AQPs* in *G. hirsutum.* AQP genes were mapped on different chromosomes in *G. hirsutum*. The segmentally duplicated genes are connected by black lines between the two relevant chromosomes.

**Supplementary Table 1** Comprehensive analysis and feature list of aquaporins in *Gossypium* genome

| **Species** | **Gene Name** | **Li et al** | **Gene ID-NAU** | **AA** | **TMD** | **MW** | **pI** | **NPA motifs** | **WOLF PSORT** | **ar/R Selectivity filter** | | | |  |
| --- | --- | --- | --- | --- | --- | --- | --- | --- | --- | --- | --- | --- | --- | --- |
|  |  |  |  |  |  |  |  |  |  | **H2** | **H5** | **LE1** | **LE2** |  |
| **AD_1_** | *GhPIP1;1_A* | *GhPIP1;4a_At* | *Gh_A03G0138* | 287 | 6 | 30852.88 | 8.6 | NPA/NPA | plasma | F | H | T | R |  |
|  | *GhPIP1;1_D* | *GhPIP1;4a_Dt* | *Gh_D03G1443* | 287 | 6 | 30852.88 | 8.6 | NPA/NPA | plasma | F | H | T | R |  |
|  | *GhPIP1;2_A* | *GhPIP1;4b_At* | *Gh_A08G1640* | 546 | 6 | 59582.10 | 8.8 | NPA/NPA | plasma | F | H | T | R |  |
|  | *GhPIP1;2_D* | *GhPIP1;4b_Dt* | *Gh_D08G1970* | 287 | 6 | 30920.88 | 8.6 | NPA/NPA | plasma | F | H | T | R |  |
|  | *GhPIP1;3_D* | *GhPIP1;4d_Dt* | *Gh_D11G0941* | 287 | 6 | 30804.83 | 8.98 | NPA/NPA | plasma | F | H | T | R |  |
|  | *GhPIP1;3_A* | *GhPIP1;4d_At* | *Gh_A11G0803* | 215 | 4 | 22695.64 | 9.73 | NPA/NPA | chloroplast | F | H | T | R |  |
|  | *GhPIP1;9_A* | *GhPIP1;4e_At* | *Gh_A11G2938* | 287 | 5 | 30969.93 | 9.1 | NPA/NPA | plasma | F | H | T | R |  |
|  | *GhPIP1;9_D* | *GhPIP1;4e_Dt* | *Gh_D11G3324* | 281 | 6 | 30297.02 | 8.96 | NPA/NPA | plasma | F | H | T | R |  |
|  | *GhPIP1;10_A* | *GhPIP1;4c_At* | *Gh_A09G1529* | 287 | 5 | 30787.75 | 9.23 | NPA/NPA | plasma | F | H | T | R |  |
|  | *GhPIP1;10_D* | *GhPIP1;4c_Dt* | *Gh_D09G1547* | 287 | 5 | 30767.76 | 9.23 | NPA/NPA | plasma | F | H | T | R |  |
|  | *GhPIP1;8_A* | *GhPIP1;4g_At* | *Gh_A13G0156* | 289 | 6 | 31055.12 | 8.63 | NPA/NPA | plasma | F | H | T | R |  |
|  | *GhPIP1;8_D* | *GhPIP1;4g_Dt* | *Gh_D13G0175* | 289 | 6 | 31089.29 | 9.21 | NPA/NPA | plasma | F | H | T | R |  |
|  | *GhPIP1;4_D* | *GhPIP1;4f_Dt* | *Gh_D06G2371* | 289 | 6 | 30867.01 | 8.81 | NPA/NPA | plasma | F | H | T | R |  |
|  | *GhPIP1;7_A* | *GhPIP1;4f_At* | *Gh_A06G1450* | 289 | 6 | 30867.01 | 8.81 | NPA/NPA | plasma | F | H | T | R |  |
|  | *GhPIP1;7_D* | *GhPIP1;4j_Dt* | *Gh_D10G2361* | 288 | 5 | 30798.84 | 8.97 | NPA/NPA | plasma | F | H | T | R |  |
|  | *GhPIP1;6_A* | *GhPIP1;4j_At* | *Gh_A10G2091* | 288 | 5 | 30764.82 | 8.97 | NPA/NPA | plasma | F | H | T | R |  |
|  | *GhPIP1;5_A* | *GhPIP1;4h_At* | *Gh_A10G2089* | 288 | 5 | 30780.82 | 8.96 | NPA/NPA | plasma | F | H | T | R |  |
|  | *GhPIP1;4_A* | *GhPIP1;4i_At* | *Gh_A10G2090* | 288 | 5 | 30780.82 | 8.96 | NPA/NPA | plasma | F | H | T | R |  |
|  | *GhPIP1;6_D* | *GhPIP1;4i_Dt* | *Gh_D10G2360* | 288 | 5 | 30766.79 | 8.96 | NPA/NPA | plasma | F | H | T | R |  |
|  | *GhPIP1;5_D* | *GhPIP1;4h_Dt* | *Gh_D10G2359* | 288 | 5 | 30762.76 | 8.81 | NPA/NPA | plasma | F | H | T | R |  |
|  | *GhPIP2;14_A* | *GhPIP2;7d_At* | *Gh_A10G0888* | 280 | 6 | 29877.87 | 9.36 | NPA/NPA | plasma | F | H | T | R |  |
|  | *GhPIP2;14_D* | *GhPIP2;7d_Dt* | *Gh_D10G0861* | 281 | 6 | 29997.06 | 9.33 | NPA/NPA | plasma | F | H | T | R |  |
|  | *GhPIP2;10_D* | *GhPIP2;8_Dt* | *Gh_D02G2248* | 278 | 6 | 29742.60 | 9 | NPA/NPA | plasma | F | H | T | R |  |
|  | *GhPIP2;10_A* | *GhPIP2;8_At* | *Gh_A03G1815* | 278 | 6 | 29723.64 | 9 | NPA/NPA | plasma | F | H | T | R |  |
|  | *GhPIP2;11_A* | *GhPIP2;7a_At* | *Gh_A01G0019* | 278 | 6 | 29558.50 | 8.82 | NPA/NPA | plasma | F | H | T | R |  |
|  | *GhPIP2;11_D* | *GhPIP2;7a_Dt* | *Gh_D01G0018* | 278 | 6 | 29581.55 | 8.82 | NPA/NPA | plasma | F | H | T | R |  |
|  | *GhPIP2;12_A* | *GhPIP2;7b_Dt* | *Gh_D01G1648* | 278 | 6 | 29680.58 | 8.99 | NPA/NPA | plasma | F | H | T | R |  |
|  | *GhPIP2;12_D* | *GhPIP2;7b_At* | *Gh_A01G1406* | 278 | 6 | 29618.47 | 8.83 | NPA/NPA | plasma | F | H | T | R |  |
|  | *GhPIP2;13_D* | *GhPIP2;7c_Dt* | *Gh_D11G1746* | 272 | 6 | 28860.76 | 9.13 | NPA/NPA | plasma | F | H | T | R |  |
|  | *GhPIP2;13_A* | *GhPIP2;7c_At* | *Gh_A11G1588* | 272 | 6 | 28835.67 | 8.94 | NPA/NPA | plasma | F | H | T | R |  |
|  | *GhPIP2;8_D* | *GhPIP2;2d_Dt* | *Gh_D08G0007* | 285 | 6 | 30581.47 | 8.2 | NPA/NPA | plasma | F | H | T | R |  |
|  | *GhPIP2;8_A* | *GhPIP2;2d_At* | *Gh_A08G2559* | 285 | 6 | 30623.55 | 8.2 | NPA/NPA | plasma | F | H | T | R |  |
|  | *GhPIP2;2_D* | *GhPIP2;4c_Dt* | *Gh_D05G0972* | 289 | 6 | 31014.00 | 7.64 | NPA/NPA | plasma | F | H | T | R |  |
|  | *GhPIP2;2_A* | *GhPIP2;4c_At* | *Gh_A05G0889* | 289 | 6 | 31020.99 | 8.2 | NPA/NPA | plasma | F | H | T | R |  |
|  | *GhPIP2;3_A* | *GhPIP2;4a_At* | *Gh_A03G0281* | 328 | 6 | 35403.00 | 6.78 | NPA/NPA | plasma | F | H | T | R |  |
|  | *GhPIP2;3_D* | *GhPIP2;4a_Dt* | *Gh_D03G1822* | 291 | 6 | 31173.11 | 6.41 | NPA/NPA | plasma | F | H | T | R |  |
|  | *GhPIP2;9_D* | *GhPIP2;2b_Dt* | *Gh_D08G2105* | 282 | 6 | 30364.29 | 7.63 | NPA/NPA | plasma | F | H | T | R |  |
|  | *GhPIP2;9_A* | *GhPIP2;2b_At* | *Gh_A08G1761* | 282 | 6 | 30338.31 | 7.63 | NPA/NPA | plasma | F | H | T | R |  |
|  | *GhPIP2;4_D* | *GhPIP2;5_Dt* | *Gh_D10G2100* | 282 | 6 | 29995.98 | 9.34 | NPA/NPA | plasma | F | H | T | R |  |
|  | *GhPIP2;4_A* | *GhPIP2;5_At* | *Gh_A10G1843* | 282 | 6 | 30023.01 | 9.34 | NPA/NPA | plasma | F | H | T | R |  |
|  | *GhPIP2;6_D* | *GhPIP2;2a_Dt* | *Gh_D01G2085* | 237 | 5 | 24924.97 | 8.78 | NPA/NPA | plasma | F | H | T | R |  |
|  | *GhPIP2;1_D* | *GhPIP2;2e_Dt* | *Gh_D01G2086* | 285 | 6 | 30451.26 | 6.57 | NPA/NPA | plasma | F | H | T | R |  |
|  | *GhPIP2;1_A* | *GhPIP2;2a_At* | *Gh_A01G1843* | 285 | 6 | 30407.25 | 6.99 | NPA/NPA | plasma | F | H | T | R |  |
|  | *GhPIP2;6_A* | *GhPIP2;2e_At* | *Gh_A01G1844* | 285 | 6 | 30363.15 | 6.99 | NPA/NPA | plasma | F | H | T | R |  |
|  | *GhPIP2;7_A* | *GhPIP2;1_At* | *Gh_A09G1405* | 285 | 6 | 30477.34 | 6.99 | NPA/NPA | plasma | F | H | T | R |  |
|  | *GhPIP2;7_D* | *GhPIP2;1_Dt* | *Gh_D09G1409* | 285 | 6 | 30489.33 | 6.99 | NPA/NPA | plasma | F | H | T | R |  |
|  | *GhPIP2;5_D* | *GhPIP2;2c_Dt* | *Gh_D04G1090* | 285 | 6 | 30290.13 | 6.99 | NPA/NPA | plasma | F | H | T | R |  |
|  | *GhPIP2;5_A* | *GhPIP2;2c_At* | *Gh_A04G0629* | 285 | 6 | 30292.14 | 6.99 | NPA/NPA | plasma | F | H | T | R |  |
|  | *-* | *GhPIP2;4b_Dt* | *Gh_D12G1974* |  |  |  |  |  |  |  |  |  |  |  |
|  | *-* | *GhPIP2;4d_Dt* | *Gh_D13G1155* |  |  |  |  |  |  |  |  |  |  |  |
|  | *GhTIP1;1_A* | *GhTIP1;1c_At* | *Gh_A04G1393* | 251 | 6 | 25748.80 | 5.36 | NPA/NPA | plasma | H | I | D | V |  |
|  | *GhTIP1;1_D* | *GhTIP1;1c_Dt* | *Gh_D04G1049* | 251 | 6 | 25748.80 | 5.36 | NPA/NPA | plasma | H | I | D | V |  |
|  | *GhTIP1;2_A* | *GhTIP1;1b_At* | *Gh_A11G2882* | 251 | 6 | 25891.96 | 4.95 | NPA/NPA | plasma | H | I | D | V |  |
|  | *GhTIP1;2_D* | *GhTIP1;1b_Dt* | *Gh_D11G3268* | 251 | 6 | 25905.94 | 4.95 | NPA/NPA | plasma | H | I | D | V |  |
|  | *GhTIP1;3_A* | *GhTIP1;1a_At* | *Gh_A01G1820* | 249 | 7 | 25512.58 | 5.89 | NPA/NPA | plasma | H | I | D | V |  |
|  | *GhTIP1;3_D* | *GhTIP1;1a_Dt* | *Gh_D01G2058* | 249 | 7 | 25509.58 | 6.12 | NPA/NPA | plasma | H | I | D | V |  |
|  | *GhTIP1;4_A* | *GhTIP1;3c_At* | *Gh_A10G1912* | 252 | 6 | 26190.31 | 5.77 | NPA/NPA | plasma | H | I | D | V |  |
|  | *GhTIP1;4_D* | *GhTIP1;3c_Dt* | *Gh_D10G2205* | 252 | 6 | 26069.16 | 5.77 | NPA/NPA | vacuole | H | I | D | V |  |
|  | *GhTIP1;5_A* | *GhTIP1;3d_At* | *Gh_A13G1995* | 252 | 6 | 25818.86 | 4.8 | NPA/NPA | vacuole | H | I | D | V |  |
|  | *GhTIP1;5_D* | *GhTIP1;3d_Dt* | *Gh_D13G2394* | 252 | 6 | 25842.88 | 4.68 | NPA/NPA | vacuole | H | I | D | V |  |
|  | *GhTIP1;6_D* | *GhTIP1;3b_Dt* | *Gh_D05G0956* | 226 | 5 | 23429.07 | 5.9 | NPA/NPA | plasma | H | I | D | V |  |
|  | *GhTIP1;6_A* | *GhTIP1;3b_At* | *Gh_A05G0834* | 252 | 7 | 25972.99 | 5.14 | NPA/NPA | plasma | H | I | D | V |  |
|  | *GhTIP1;7_A* | *GhTIP1;3a_At* | *Gh_A03G0325* | 252 | 7 | 25836.88 | 5.14 | NPA/NPA | plasma | H | I | D | V |  |
|  | *GhTIP1;7_D* | *GhTIP1;3a_Dt* | *Gh_D03G1253* | 252 | 7 | 25836.88 | 5.14 | NPA/NPA | plasma | H | I | D | V |  |
|  | *GhTIP2;2_A* | *GhTIP2;3a_At* | *Gh_A03G0083* | 251 | 7 | 25162.30 | 5.03 | NPA/NPA | vacuole | H | I | S | R |  |
|  | *GhTIP2;2_D* | *GhTIP2;3a_Dt* | *Gh_D03G1568* | 250 | 6 | 25002.11 | 5.03 | NPA/NPA | vacuole | H | I | S | R |  |
|  | *GhTIP2;3_D* | *GhTIP2;3b_Dt* | *Gh_D08G1823* | 258 | 6 | 25749.80 | 4.59 | NPA/NPA | vacuole | H | I | S | R |  |
|  | *GhTIP2;3_A* | *GhTIP2;3b_At* | *Gh_A08G1523* | 250 | 6 | 25102.25 | 5.37 | NPA/NPA | vacuole | H | I | S | R |  |
|  | *GhTIP2;1_A* | *GhTIP2;1_At* | *Gh_A02G1800* | 248 | 7 | 25079.20 | 5.31 | NPA/NPA | vacuole | H | I | S | R |  |
|  | *GhTIP2;1_D* | *GhTIP2;1_Dt* | *Gh_D03G0583* | 248 | 7 | 25049.18 | 5.31 | NPA/NPA | vacuole | H | I | S | R |  |
|  | *GhTIP3;1_A* | *GhTIP3;2_At* | *Gh_A03G2098* | 256 | 6 | 27066.64 | 7.92 | NPA/NPA | plasma | H | I | D | R |  |
|  | *GhTIP3;1_D* | *GhTIP3;2_Dt* | *Gh_D02G1191* | 256 | 6 | 27112.73 | 7.84 | NPA/NPA | plasma | H | I | D | R |  |
|  | *GhTIP4;1_A* | *GhTIP4;1_At* | *Gh_A06G0845* | 181 | 5 | 18553.02 | 5.86 | NPA/ | vacuole | H | S |  |  |  |
|  | *GhTIP4;1_D* | *GhTIP4;1_Dt* | *Gh_D06G0982* | 269 | 6 | 28395.98 | 5.48 | NPA/NPA | plasma | H | I | S | R |  |
|  | *GhTIP5;1_A* | *GhTIP5;1_At* | *Gh_A07G0358* | 253 | 6 | 26146.36 | 8.73 | NPA/NPA | plasma | N | V | S | L |  |
|  | *GhTIP5;1_D* | *GhTIP5;1_Dt* | *Gh_D07G0420* | 253 | 6 | 26023.21 | 7.86 | NPA/NPA | plasma | N | V | S | L |  |
|  | *GhNIP1;3_D* | *GhNIP1;2c_Dt* | *Gh_D10G0313* | 280 | 6 | 29620.51 | 8.97 | NPA/NPA | plasma | W | V | T | R |  |
|  | *GhNIP1;3_A* | *GhNIP1;2c_At* | *Gh_A10G0307* | 280 | 6 | 29678.45 | 8.98 | NPA/NPA | plasma | W | V | T | R |  |
|  | *GhNIP1;2_A* | *GhNIP1;2a_At* | *Gh_A06G1128* | 275 | 5 | 29208.90 | 8.76 | NPA/NPA | plasma | W | V | T | R |  |
|  | *GhNIP1;2_D* | *GhNIP1;2a_Dt* | *Gh_D06G1464* | 275 | 5 | 29226.87 | 8.76 | NPA/NPA | plasma | W | V | T | R |  |
|  | *GhNIP1;1_D* | *GhNIP1;2b_Dt* | *Gh_D06G2131* | 270 | 6 | 28984.49 | 5.82 | NPA/NPA | plasma | W | V | T | R |  |
|  | *GhNIP1;1_A* | *GhNIP1;2b_At* | *Gh_A06G1993* | 270 | 6 | 28960.50 | 6.04 | NPA/NPA | plasma | W | V | T | R |  |
|  | *GhNIP2;1_D* | *GhNIP4;1_Dt* | *Gh_D02G1465* | 269 | 6 | 29032.50 | 8.90 | NPA/NPA | vacuole | W | V | S | R |  |
|  | *GhNIP2;1_A* | *GhNIP4;1_At* | *Gh_A03G1055* | 269 | 6 | 28892.27 | 7.64 | NPA/NSA | vacuole | W | V | S | R |  |
|  | *GhNIP3;1_A* | *GhNIP1;1_At* | *Gh_A05G1940* | 292 | 6 | 31553.93 | 6.71 | NPA/NPA | plasma | W | V | T | R |  |
|  | *GhNIP3;1_D* | *GhNIP1;1_Dt* | *Gh_D05G2171* | 365 | 7 | 39129.93 | 6.36 | NPA/NPA | plasma | W | V | T | R |  |
|  | *GhNIP5;1_D* | *GhNIP5;1_Dt* | *Gh_D08G1149* | 298 | 5 | 31116.01 | 8.57 | NPS/NPV | plasma | A | I | S | R |  |
|  | *GhNIP5;1_A* | *GhNIP5;1_At* | *Gh_A08G0940* | 298 | 5 | 31146.04 | 8.57 | NPS/NPV | plasma | A | I | S | R |  |
|  | *GhNIP6;1_A* | *GhNIP6;1_At* | *Gh_A05G1025* | 183 | 5 | 19006.42 | 9.51 | NPT/NPV | plasma |  | I | T | R |  |
|  | *GhNIP6;1_D* | *GhNIP6;1_Dt* | *Gh_D05G1142* | 300 | 6 | 30974.16 | 8.99 | NPA/NPV | plasma | T | I | T | R |  |
|  | *GhNIP4;1_D* | *GhNIP4;2_Dt* | *Gh_D11G0733* | 268 | 5 | 28301.79 | 9.01 | NPA/NPA | plasma | G | S | S | R |  |
|  | *GhNIP4;1_A* | *GhNIP4;2_At* | *Gh_A11G3043* | 268 | 5 | 28289.78 | 9.03 | NPA/NPA | plasma | G | S | S | R |  |
|  | *GhNIP7;2_A* | *GhNIP7;1b_At* | *Gh_A09G2266* | 192 | 4 | 20179.47 | 6.02 | NPA | vacuole |  | V | S | R |  |
|  | *GhNIP7;2_D* | *GhNIP7;1b_Dt* | *Gh_D09G2063* | 266 | 7 | 27793.56 | 5.46 | NPA/NPA | vacuole | A | V | S | R |  |
|  | *GhNIP7;1_A* | *GhNIP7;1a_At* | *Gh_A09G0535* | 209 | 6 | 21908.19 | 9.82 | NPA/NPA | vacuole | A | V | S | R |  |
|  | *GhNIP7;1_D* | *GhNIP7;1a_Dt* | *Gh_D09G0532* | 209 | 6 | 21914.17 | 9.82 | NPA/NPA | vacuole | A | V | S | R |  |
|  | *GhSIP1;4_D* | *GhSIP1;1a_Dt* | *Gh_D05G2080* | 241 | 6 | 26245.29 | 9.71 | NPT/NPA | extracellular | V | V | T | N |  |
|  | *GhSIP1;3_D* | *GhSIP1;1b_Dt* | *Gh_D04G0138* | 241 | 5 | 26003.90 | 9.68 | NPT/NPA | vacuole | V | V | T | N |  |
|  | *GhSIP1;3_A* | *GhSIP1;1b_At* | *Gh_A07G2010* | 241 | 5 | 26238.23 | 9.81 | NPT/NPA | vacuole | V | V | T | N |  |
|  | *GhSIP1;5_D* | *GhSIP1;1d_Dt* | *Gh_D10G1912* | 243 | 3 | 25980.92 | 10.15 | NPA/NPA | vacuole | V | V | T | S |  |
|  | *GhSIP1;5_A* | *GhSIP1;1d_At* | *Gh_A10G1656* | 243 | 3 | 25909.82 | 10.1 | NPA/NPA | vacuole | V | V | T | S |  |
|  | *GhSIP1;2_D* | *GhSIP1;1c_Dt* | *Gh_D13G1761* | 759 | 7 | 82359.49 | 7.18 | DPA/NPA | plasma | F | V | T | F |  |
|  | *GhSIP1;2_A* | *GhSIP1;1c_At* | *Gh_A13G1436* | 240 | 5 | 25962.64 | 8.87 | DPA/NPA | plasma | F | V | T | F |  |
|  | *GhSIP1;1_D* | *GhSIP1;2_Dt* | *Gh_D11G2472* | 247 | 5 | 26905.93 | 9.63 | NPT/NPA | vacuole | I | V | T | N |  |
|  | *GhSIP1;1_A* | *GhSIP1;2_At* | *Gh_A11G2172* | 247 | 5 | 26796.87 | 9.63 | NPT/NPA | vacuole | I | V | T | N |  |
|  | *GhSIP2;1_D* | *GhSIP2;1_Dt* | *Gh_D05G3636* | 236 | 6 | 25695.53 | 9.78 | NPL/NPA | vacuole | V | H | T | S |  |
|  | *GhSIP2;1_A* | *GhSIP2;1_At* | *Gh_A04G0089* | 236 | 6 | 25566.31 | 9.68 | NPL/NPA | vacuole | V | H | T | S |  |
|  | *GhXIP1;1_A* | *GhXIP1;1_At* | *Gh_A01G1351* | 303 | 6 | 32234.05 | 8.22 | NPI/NPA | plasma | F | V | G | R |  |
|  | *GhXIP1;1_D* | *GhXIP1;1_Dt* | *Gh_D01G1596* | 303 | 6 | 32291.15 | 7.65 | NPI/NPA | plasma | F | V | G | R |  |
|  | *GhXIP1;2_D* | *GhXIP2;2_Dt* | *Gh_D12G0296* | 267 | 6 | 28438.65 | 8.56 | NPV/NPA | plasma | V | V | A | R |  |
|  | *GhXIP1;2_A* | *GhXIP2;2_At* | *Gh_A12G0369* | 295 | 7 | 31427.08 | 7.62 | NPV/NPA | plasma | V | V | A | R |  |
|  | *GhXIP2;1_A* | *GhXIP2;1_At* | *Gh_A12G0370* | 305 | 5 | 32552.92 | 8.57 | NPA | plasma | L | V | A | R |  |
|  | *GhXIP2;1_D* | *GhXIP2;1_Dt* | *Gh_D12G0295* | 278 | 6 | 29466.88 | 8.78 | SPV/NPA | vacuole | I | V | A | R |  |
| A_2_ | *GaPIP1;1* | *GaPIP1;4a* | *Ga01G2570* | 287 | 6 | 30852.88 | 8.6 | NPA/NPA | plasma |  |  |  |  |  |
|  | *GaPIP1;2* | *GaPIP1;4b* | *Ga08G2153* | 287 | 6 | 30906.85 | 8.6 | NPA/NPA | plasma |  |  |  |  |  |
|  | *GaPIP1;3* | *GaPIP1;4c* | *Ga09G1963* | 261 | 5 | 28169.56 | 8.87 | NPA/NPA | plasma |  |  |  |  |  |
|  | *GaPIP1;4* | *GaPIP1;4d* | *Ga11G3124* | 287 | 5 | 30652.64 | 8.81 | NPA/NPA | plasma |  |  |  |  |  |
|  | *GaPIP1;5* | *GaPIP1;4e* | *Ga11G0022* | 288 | 5 | 30780.82 | 8.96 | NPA/NPA | plasma |  |  |  |  |  |
|  | *GaPIP1;6* | *GaPIP1;4f* | *Ga06G2010* | 288 | 5 | 30764.82 | 8.97 | NPA/NPA | plasma |  |  |  |  |  |
|  | *GaPIP1;7* | *GaPIP1;4g* | *Ga13G0191* | 289 | 6 | 30867.01 | 8.81 | NPA/NPA | plasma |  |  |  |  |  |
|  | *GaPIP1;8* | *GaPIP1;4h* | *Ga10G0006* | 289 | 6 | 31055.12 | 8.63 | NPA/NPA | plasma |  |  |  |  |  |
|  | *GaPIP1;9* | *GaPIP1;4i* | *Ga10G0007* | 287 | 5 | 30953.89 | 8.97 | NPA/NPA | plasma |  |  |  |  |  |
|  | *GaPIP1;10* | *GaPIP2;1* | *Ga09G1708* | 366 | 6 | 39624.90 | 9.03 | NPA/NPA | chloroplast |  |  |  |  |  |
|  | *GaPIP2;1* | *GaPIP2;2a* | *Ga02G1659* | 291 | 6 | 31215.13 | 6.51 | NPA/NPA | plasma |  |  |  |  |  |
|  | *GaPIP2;2* | *GaPIP2;2b* | *Ga08G2320* | 289 | 6 | 31020.99 | 8.2 | NPA/NPA | plasma |  |  |  |  |  |
|  | *GaPIP2;3* | *GaPIP2;2c* | *Ga04G0829* | 285 | 6 | 30623.55 | 8.2 | NPA/NPA | plasma |  |  |  |  |  |
|  | *GaPIP2;4* | *GaPIP2;2d* | *Ga08G0016* | 282 | 6 | 30324.28 | 7.63 | NPA/NPA | plasma |  |  |  |  |  |
|  | *GaPIP2;5* | *GaPIP2;4a* | *Ga01G2379* | 285 | 6 | 30282.15 | 7.65 | NPA/NPA | plasma |  |  |  |  |  |
|  | *GaPIP2;6* | *GaPIP2;4c* | *Ga05G1091* | 285 | 6 | 30438.26 | 6.58 | NPA/NPA | plasma |  |  |  |  |  |
|  | *GaPIP2;7* | *GaPIP2;5a* | *Ga10G0547* | 285 | 6 | 30425.29 | 6.99 | NPA/NPA | plasma |  |  |  |  |  |
|  | *GaPIP2;8* | *GaPIP2;7a* | *Ga01G0023* | 198 | 5 | 20956.52 | 9.85 | NPA/NPA | plasma |  |  |  |  |  |
|  | *GaPIP2;10* | *GaPIP2;7b* | *Ga02G1129* | 311 | 6 | 33225.76 | 9.06 | NPA/NPA | chloroplast |  |  |  |  |  |
|  | *GaPIP2;11* | *GaPIP2;7c* | *Ga11G2179* | 278 | 6 | 29558.50 | 8.82 | NPA/NPA | plasma |  |  |  |  |  |
|  | *GaPIP2;12* | *GaPIP2;7d* | *Ga10G1988* | 275 | 6 | 29361.18 | 8.83 | NPA/NPA | plasma |  |  |  |  |  |
|  | *GaPIP2;13* | *GaPIP2;8* | *Ga03G2644* | 272 | 6 | 28905.81 | 8.94 | NPA/NPA | plasma |  |  |  |  |  |
|  | *GaPIP2;14* | *-* | *Ga05G1345* | 280 | 6 | 29891.89 | 9.36 | NPA/NPA | cytoplasm |  |  |  |  |  |
|  | *GaTIP1;1* | *GaTIP1;1a* | *Ga02G1634* | 251 | 6 | 25748.80 | 5.36 | NPA/NPA | cytoplasm |  |  |  |  |  |
|  | *GaTIP1;2* | *GaTIP1;1b* | *Ga11G0084* | 251 | 6 | 25891.96 | 4.95 | NPL/NPA | cytoplasm |  |  |  |  |  |
|  | *GaTIP1;3* | *GaTIP1;1c* | *Ga04G0887* | 249 | 7 | 25512.58 | 5.89 | NPA/NPA | cytoplasm |  |  |  |  |  |
|  | *GaTIP1;4* | *GaTIP1;3a* | *Ga01G2333* | 250 | 7 | 25976.03 | 5.77 | NPA/NPA | cytoplasm |  |  |  |  |  |
|  | *GaTIP1;5* | *GaTIP1;3b* | *Ga05G1034* | 252 | 6 | 25801.87 | 4.8 | NPA/NPA | vacuole |  |  |  |  |  |
|  | *GaTIP1;6* | *GaTIP1;3c* | *Ga10G0412* | 252 | 7 | 25972.99 | 5.14 | NPA/NPA | cytoplasm |  |  |  |  |  |
|  | *GaTIP1;7* | *GaTIP1;3d* | *Ga13G2780* | 252 | 7 | 25836.88 | 5.14 | NPA/NPA | cytoplasm |  |  |  |  |  |
|  | *GaTIP2;1* | *GaTIP2;1* | *Ga02G0652* | 248 | 7 | 25063.21 | 5.31 | NPA/NPA | vacuole |  |  |  |  |  |
|  | *GaTIP2;2* | *GaTIP2;3a* | *Ga01G2805* | 250 | 6 | 25025.15 | 5.03 | NPA/NPA | vacuole |  |  |  |  |  |
|  | *GaTIP2;3* | *GaTIP2;3b* | *Ga08G1979* | 250 | 6 | 25160.29 | 5.07 | NPA/NPA | vacuole |  |  |  |  |  |
|  | *GaTIP3;1* | *GaTIP3;2* | *Ga03G1256* | 256 | 6 | 27118.69 | 7.92 | NPA/NPA | cytoplasm |  |  |  |  |  |
|  | *GaTIP4;1* | *GaTIP4;1* | *Ga06G1055* | 246 | 7 | 25702.19 | 5.91 | NPA/NPA | vacuole |  |  |  |  |  |
|  | *GaTIP5;1* | *GaTIP5;1* | *Ga07G0505* | 253 | 6 | 26173.43 | 9.1 | NPA/NPA | chloroplast |  |  |  |  |  |
|  | *GaNIP1;1* | *GaNIP1;1* | *Ga05G2402* | 195 | 4 | 20999.07 | 5.22 | NPA/NPA | plasma |  |  |  |  |  |
|  | *GaNIP1;2* | *GaNIP1;2a* | *Ga11G1349* | 275 | 5 | 29185.86 | 8.76 | NPA/NPA | plasma |  |  |  |  |  |
|  | *GaNIP1;3* | *GaNIP1;2b* | *Ga06G2445* | 280 | 6 | 29615.37 | 8.98 | NPA/NPA | plasma |  |  |  |  |  |
|  | *GaNIP2;1* | *GaNIP1;2c* | *Ga10G2732* | 261 | 6 | 28049.38 | 8.68 | NPA/NPA | vacuole |  |  |  |  |  |
|  | *GaNIP3;1* | *GaNIP4;1* | *Ga03G1630* | 292 | 6 | 31544.96 | 7.7 | NPA/NPA | plasma |  |  |  |  |  |
|  | *GaNIP4;1* | *GaNIP4;2* | *Ga11G3352* | 268 | 5 | 28363.85 | 9.12 | NPA/NPA | plasma |  |  |  |  |  |
|  | *GaNIP6;1* | *GaNIP5;1* | *Ga08G1251* | 300 | 6 | 31020.25 | 8.99 | NPS/NPV | plasma |  |  |  |  |  |
|  | *GaNIP7;1* | *GaNIP6;1* | *Ga05G1268* | 274 | 6 | 29178.45 | 9.51 | NPA/NPV | plasma |  |  |  |  |  |
|  | *GaNIP7;2* | *GaNIP7;1a* | *Ga09G0766* | 290 | 6 | 30759.71 | 5.81 | NPA/NPA | plasma |  |  |  |  |  |
|  | *-* | *GaNIP7;1b* | *Ga09G2454* |  |  |  |  |  |  |  |  |  |  |  |
|  | *GaSIP1;1* | *GaSIP1;1b* | *Ga07G2544* | 247 | 5 | 26873.00 | 9.52 | NPA/NPA | vacuole |  |  |  |  |  |
|  | *GaSIP1;2* | *GaSIP1;1c* | *Ga13G2068* | 240 | 6 | 25937.55 | 7.78 | NPA/NPA | plasma |  |  |  |  |  |
|  | *GaSIP1;3* | *GaSIP1;1d* | *Ga10G0848* | 240 | 5 | 26125.15 | 9.81 | NPT/NPA | chloroplast |  |  |  |  |  |
|  | *GaSIP1;4* | *GaSIP1;2* | *Ga11G1233* | 173 | 3 | 19190.76 | 9.74 | DPA/NPA | vacuole |  |  |  |  |  |
|  | *GaSIP1;5* | *GaSIP2;1* | *Ga05G4144* | 243 | 3 | 25906.80 | 10.15 | NPT/NPA | vacuole |  |  |  |  |  |
|  | *GaSIP2;1* | - |  | 243 | 3 | 25533.23 | 9.58 | NPT/NPA | vacuole |  |  |  |  |  |
|  | *GaXIP1;1* | *GaXIP1;1* | *Ga02G1057* | 303 | 6 | 32280.08 | 8.22 | NPA/NPA | plasma |  |  |  |  |  |
|  | *GaXIP1;2* | *GaXIP2;1* | *Ga14G1867* | 307 | 7 | 32734.58 | 7.63 | NPA/NPA | vacuole |  |  |  |  |  |
|  | *GaXIP2;1* | *GaXIP2;2* | *Ga14G1866* | 278 | 6 | 29443.00 | 8.87 | NPI/NPA | vacuole |  |  |  |  |  |
| D_5_ | *GrPIP1;1* | *GrPIP1;4a* | *Gorai.006G181300* | 287 | 6 | 30852.88 | 8.6 | NPA/NPA | plasma |  |  |  |  |  |
|  | *GrPIP1;2* | *GrPIP1;4c* | *Gorai.007G100000* | 287 | 6 | 30920.88 | 8.6 | NPA/NPA | plasma |  |  |  |  |  |
|  | *GrPIP1;3* | *GrPIP1;4d* | *Gorai.007G378400* | 286 | 6 | 21783.56 | 9.61 | NPA/NPA | plasma |  |  |  |  |  |
|  | *GrPIP1;4* | *GrPIP1;4e* | *Gorai.010G198800* | 289 | 6 | 30895.07 | 8.97 | NPA/NPA | plasma |  |  |  |  |  |
|  | *GrPIP1;5* | *GrPIP1;4f* | *Gorai.013G019300* | 288 | 5 | 30776.83 | 8.96 | NPA/NPA | plasma |  |  |  |  |  |
|  | *GrPIP1;6* | *GrPIP1;4g* | *Gorai.011G283200* | 288 | 5 | 30766.79 | 8.96 | NPA/NPA | plasma |  |  |  |  |  |
|  | *GrPIP1;7* | *GrPIP1;4h* | *Gorai.011G283300* | 288 | 5 | 30798.84 | 8.97 | NPA/NPA | plasma |  |  |  |  |  |
|  | *GrPIP1;8* | *GrPIP1;4i* | *Gorai.011G283400* | 289 | 6 | 31103.32 | 9.21 | NPA/NPA | plasma |  |  |  |  |  |
|  | *GrPIP1;9* | *GrPIP1;4j* | *Gorai.004G212800* | 287 | 5 | 30863.81 | 9.1 | NPA/NPA | plasma |  |  |  |  |  |
|  | *GrPIP1;10* | *GrPIP1;4b* | *Gorai.006G166800* | 287 | 5 | 30767.76 | 9.23 | NPA/NPA | plasma |  |  |  |  |  |
|  | *GrPIP2;1* | *GrPIP2;1* | *Gorai.008G216300* | 285 | 6 | 30407.25 | 6.99 | NPA/NPA | plasma |  |  |  |  |  |
|  | *GrPIP2;2* | *GrPIP2;4b* | *Gorai.009G107200* | 285 | 6 | 30425.29 | 6.99 | NPA/NPA | plasma |  |  |  |  |  |
|  | *GrPIP2;3* | *GrPIP2;4c* | *Gorai.013G127300* | 285 | 6 | 30290.13 | 6.99 | NPA/NPA | plasma |  |  |  |  |  |
|  | *GrPIP2;4* | *GrPIP2;4d* | *Gorai.011G239500* | 282 | 6 | 29981.96 | 9.34 | NPA/NPA | plasma |  |  |  |  |  |
|  | *GrPIP2;5* | *GrPIP2;5a* | *Gorai.011G253400* | 245 | 6 | 25805.30 | 9.57 | NPA/NPA | plasma |  |  |  |  |  |
|  | *GrPIP2;6* | *GrPIP2;5b* | *Gorai.002G002500* | 320 | 6 | 34568.96 | 6.78 | NPA/NPA | plasma |  |  |  |  |  |
|  | *GrPIP2;7* | *GrPIP2;7a* | *Gorai.002G198900* | 289 | 6 | 31001.95 | 7.64 | NPA/NPA | plasma |  |  |  |  |  |
|  | *GrPIP2;8* | *GrPIP2;7b* | *Gorai.007G191600* | 285 | 6 | 30581.47 | 8.2 | NPA/NPA | plasma |  |  |  |  |  |
|  | *GrPIP2;9* | *GrPIP2;7c* | *Gorai.002G248400* | 282 | 6 | 30393.33 | 8.21 | NPA/NPA | plasma |  |  |  |  |  |
|  | *GrPIP2;10* | *GrPIP2;2a* | *Gorai.004G229000* | 278 | 6 | 29742.60 | 9.00 | NPA/NPA | plasma |  |  |  |  |  |
|  | *GrPIP2;11* | *GrPIP2;2b* | *Gorai.009G418100* | 278 | 6 | 29609.60 | 8.82 | NPA/NPA | plasma |  |  |  |  |  |
|  | *GrPIP2;12* | *GrPIP2;2c* | *Gorai.004G001400* | 336 | 7 | 36470.67 | 8.6 | NPA/NPA | plasma |  |  |  |  |  |
|  | *GrPIP2;13* | *GrPIP2;2d* | *Gorai.003G141600* | 272 | 6 | 28846.73 | 9.11 | NPA/NPA | plasma |  |  |  |  |  |
|  | *GrPIP2;14* | *GrPIP2;4a* | *Gorai.002G245900* | 280 | 6 | 29925.98 | 9.33 | NPA/NPA | plasma |  |  |  |  |  |
|  | *-* | *GrPIP2;7d* | *Gorai.011G098100* |  |  |  |  |  |  |  |  |  |  |  |
|  | *-* | *GrPIP2;8* | *Gorai.005G254400* |  |  |  |  |  |  |  |  |  |  |  |
|  | *GrTIP1;1* | *GrTIP1;1a* | *Gorai.007G372700* | 251 | 6 | 25748.80 | 5.36 | NPA/NPA | cytoplasm |  |  |  |  |  |
|  | *GrTIP1;2* | *GrTIP1;1b* | *Gorai.009G413000* | 249 | 7 | 25486.54 | 6.02 | NPA/NPA | cytoplasm |  |  |  |  |  |
|  | *GrTIP1;3* | *GrTIP1;1c* | *Gorai.003G136600* | 251 | 6 | 25905.94 | 4.95 | NPA/NPA | cytoplasm |  |  |  |  |  |
|  | *GrTIP1;4* | *GrTIP1;3a* | *Gorai.009G101700* | 252 | 7 | 25972.99 | 5.14 | NPA/NPA | cytoplasm |  |  |  |  |  |
|  | *GrTIP1;5* | *GrTIP1;3b* | *Gorai.011G253100* | 252 | 7 | 25836.88 | 5.14 | NPA/NPA | cytoplasm |  |  |  |  |  |
|  | *GrTIP1;6* | *GrTIP1;3c* | *Gorai.013G265400* | 252 | 6 | 25784.84 | 4.8 | NPA/NPA | vacuole |  |  |  |  |  |
|  | *GrTIP1;7* | *GrTIP1;3d* | *Gorai.003G064000* | 252 | 6 | 26078.17 | 5.9 | NPA/NPA | cytoplasm |  |  |  |  |  |
|  | *GrTIP2;1* | *GrTIP2;1* | *Gorai.003G171800* | 248 | 7 | 25049.18 | 5.31 | NPA/NPA | vacuole |  |  |  |  |  |
|  | *GrTIP2;2* | *GrTIP2;3a* | *Gorai.004G197600* | 250 | 6 | 25029.14 | 5.03 | NPA/NPA | vacuole |  |  |  |  |  |
|  | *GrTIP2;3* | *GrTIP2;3b* | *Gorai.005G135200* | 250 | 6 | 25072.22 | 5.37 | NPA/NPA | vacuole |  |  |  |  |  |
|  | *GrTIP3;1* | *GrTIP3;2* | *Gorai.010G107900* | 256 | 6 | 27066.64 | 7.92 | NPA/NPA | cytoplasm |  |  |  |  |  |
|  | *GrTIP4;1* | *GrTIP4;1* | *Gorai.001G049000* | 246 | 6 | 25717.16 | 5.91 | NPA/NPA | vacuole |  |  |  |  |  |
|  | *GrTIP5;1* | *GrTIP5;1* | *Gorai.009G236600* | 253 | 6 | 26023.21 | 7.86 | NPA/NPA | chloroplast |  |  |  |  |  |
|  | *GrNIP1;1* | *GrNIP1;1* | *Gorai.010G162400* | 248 | 6 | 26525.92 | 8.56 | NPA/NPA | plasma |  |  |  |  |  |
|  | *GrNIP1;2* | *GrNIP1;2a* | *Gorai.010G240400* | 275 | 5 | 29226.87 | 8.76 | NPA/NPA | plasma |  |  |  |  |  |
|  | *GrNIP1;3* | *GrNIP1;2b* | *Gorai.011G035800* | 280 | 6 | 29654.52 | 8.97 | NPA/NPA | plasma |  |  |  |  |  |
|  | *GrNIP2;1* | *GrNIP1;2c* | *Gorai.005G161900* | 269 | 6 | 28923.33 | 8.67 | NPA/NPA | vacuole |  |  |  |  |  |
|  | *GrNIP3;1* | *GrNIP4;1* | *Gorai.007G078800* | 292 | 6 | 31479.79 | 6.71 | NPA/NPA | plasma |  |  |  |  |  |
|  | *GrNIP4;1* | *GrNIP4;2* | *Gorai.004G126900* | 268 | 5 | 28315.82 | 9.01 | NPA/NPA | plasma |  |  |  |  |  |
|  | *GrNIP5;1* | *GrNIP5;1* | *Gorai.009G124500* | 298 | 5 | 31130.04 | 8.57 | NPS/NPV | plasma |  |  |  |  |  |
|  | *GrNIP6;1* | *GrNIP6;1* | *Gorai.006G052300* | 300 | 6 | 30974.16 | 8.99 | NPA/NPV | plasma |  |  |  |  |  |
|  | *GrNIP7;1* | *GrNIP7;1a* | *Gorai.006G236300* | 209 | 6 | 21942.22 | 9.82 | NPA/NPA | vacuole |  |  |  |  |  |
|  | *GrNIP7;2* | *GrNIP7;1b* | *Gorai.009G226500* | 297 | 6 | 31585.82 | 6.28 | NPA/NPA | plasma |  |  |  |  |  |
|  | *GrSIP1;1* | *GrSIP1;1a* | *Gorai.012G017700* | 247 | 5 | 26905.93 | 9.63 | NPT/NPA | vacuole |  |  |  |  |  |
|  | *GrSIP1;2* | *GrSIP1;1b* | *Gorai.013G191500* | 240 | 6 | 17631.57 | 7.14 | DPA/NPA | plasma |  |  |  |  |  |
|  | *GrSIP1;3* | *GrSIP1;1c* | *Gorai.011G214600* | 241 | 5 | 26008.99 | 9.81 | NPT/NPA | vacuole |  |  |  |  |  |
|  | *GrSIP1;4* | *GrSIP1;1d* | *Gorai.007G268300* | 241 | 6 | 26242.18 | 9.38 | NPT/NPA | chloroplast |  |  |  |  |  |
|  | *GrSIP1;5* | *GrSIP1;2* | *Gorai.009G445300* | 243 | 3 | 25966.90 | 10.15 | NPA/NPA | vacuole |  |  |  |  |  |
|  | *GrSIP2;1* | *GrSIP2;1* | *Gorai.002G192500* | 236 | 6 | 25695.53 | 9.78 | NPL/NPA | vacuole |  |  |  |  |  |
|  | *GrXIP1;1* | *GrXIP1;1* | *Gorai.008G033000* | 303 | 6 | 32221.05 | 8.22 | NPI/NPA | plasma |  |  |  |  |  |
|  | *GrXIP1;2* | *GrXIP2;1* | *Gorai.008G033100* | 302 | 7 | 32307.15 | 8.17 | NPV/NPA | vacuole |  |  |  |  |  |
|  | *GrXIP2;1* | *GrXIP2;2* | *Gorai.008G033100* | 278 | 6 | 29492.97 | 8.78 | SPV/NPA | vacuole |  |  |  |  |  |

**Supplementary Table 2** Classification of aquaporins in three *Gossypium* species

| **Genome** | **PIP** | **TIP** | **NIP** | **SIP** | **XIP** | **AQP** |
| --- | --- | --- | --- | --- | --- | --- |
| **AD_1_** | 48 | 28 | 20 | 11 | 1 | 111 |
| **At** | 24 | 13 | 10 | 5 | 3 | 55 |
| **Dt** | 25 | 13 | 10 | 6 | 3 | 57 |
| **X^¶^** | **-** | 3 | - | - | 1 | 4 |
| **A_2_** | 23 | 13 | 9 | 6 | 4 | 54 |
| **D_5_** | 24 | 13 | 10 | 6 | 3 | 56 |
| **AD_1_-specific** | 1 | 0 | 1 | 1 | 0 | 3 |
|  | *GhPIP2;9_A* | | *GhNIP5;1_A* | *GhSIP2;1_A* | |  |
| **A_2_-specific** |  |  |  | *2* |  | 2 |
|  |  |  |  | *GaSIP1;4* |  |  |
|  |  |  |  | *GaSIP1;6* |  |  |

PIP, plasma membrane intrinsic protein; TIP, tonoplast intrinsic protein; NIP, nodulin-26 like intrinsic protein; SIP, small intrinsic protein; XIP, uncharacterized xintrinsic protein; AQP, aquaporin. ¶ See Supplementary Table 1 for detail.

***Supplementary Table 3*** *The identity and Ka/Ks ratio of duplicated genes in* Gossypium

| **Species** | **Duplicated gene 1** | **Duplicated gene 2** | **Identity (%)** | ***Ka*** | ***Ks*** | ***Ka/Ks*** | **Purifying selection** | **Duplicate type** |
| --- | --- | --- | --- | --- | --- | --- | --- | --- |
| **AD_1_** | *GhPIP2;14_D* | *GhPIP2;14_A* | 97.870 | 0.018 | 0.019 | 0.938 | Yes | Segmental duplication |
|  | *GhXIP2;1_D* | *GhXIP2;1_A* | 98.910 | 0.051 | 0.058 | 0.880 | Yes | Segmental duplication |
|  | *GhPIP1;8_D* | *GhPIP1;8_A* | 97.700 | 0.022 | 0.029 | 0.756 | Yes | Segmental duplication |
|  | *GhPIP2;6_A* | *GhPIP2;1_D* | 98.830 | 0.011 | 0.015 | 0.748 | Yes | Segmental duplication |
|  | *GhPIP1;2_D* | *GhPIP1;2_A* | 98.990 | 0.116 | 0.185 | 0.626 | Yes | Segmental duplication |
|  | *GhSIP1;4_D* | *GhSIP1;3_D* | 95.590 | 0.039 | 0.067 | 0.572 | Yes | Segmental duplication |
|  | *GhTIP1;6_D* | *GhTIP1;6_A* | 98.960 | 0.037 | 0.065 | 0.567 | Yes | Segmental duplication |
|  | *GhNIP2;1_D* | *GhNIP2;1_A* | 98.150 | 0.015 | 0.030 | 0.497 | Yes | Segmental duplication |
|  | *GhTIP2;3_D* | *GhTIP2;3_A* | 99.190 | 0.014 | 0.028 | 0.488 | Yes | Segmental duplication |
|  | *GhPIP2;6_D* | *GhPIP2;6_A* | 98.700 | 0.011 | 0.023 | 0.485 | Yes | Segmental duplication |
|  | *GhSIP1;3_D* | *GhSIP1;3_A* | 95.320 | 0.039 | 0.080 | 0.484 | Yes | Segmental duplication |
|  | *GhTIP5;1_D* | *GhTIP5;1_A* | 98.820 | 0.009 | 0.020 | 0.451 | Yes | Segmental duplication |
|  | *GhPIP2;13_D* | *GhPIP2;13_A* | 98.410 | 0.012 | 0.030 | 0.389 | Yes | Segmental duplication |
|  | *GhSIP1;4_D* | *GhSIP1;3_A* | 94.630 | 0.039 | 0.110 | 0.360 | Yes | Segmental duplication |
|  | *GhSIP1;1_D* | *GhSIP1;1_A* | 97.840 | 0.016 | 0.046 | 0.348 | Yes | Segmental duplication |
|  | *GhPIP1;3_D* | *GhPIP1;3_A* | 99.690 | 0.002 | 0.006 | 0.339 | Yes | Segmental duplication |
|  | *GhNIP4;1_D* | *GhNIP4;1_A* | 98.020 | 0.013 | 0.040 | 0.336 | Yes | Segmental duplication |
|  | *GhPIP2;3_D* | *GhPIP2;3_A* | 99.320 | 0.005 | 0.014 | 0.329 | Yes | Segmental duplication |
|  | *GhPIP2;6_D* | *GhPIP2;1_D* | 99.710 | 0.004 | 0.012 | 0.324 | Yes | Tandem duplication |
|  | *GhPIP2;1_D* | *GhPIP2;1_A* | 99.300 | 0.005 | 0.015 | 0.319 | Yes | Segmental duplication |
|  | *GhSIP2;1_D* | *GhSIP2;1_A* | 98.030 | 0.013 | 0.043 | 0.306 | Yes | Segmental duplication |
|  | *GhNIP1;3_D* | *GhNIP1;3_A* | 98.460 | 0.010 | 0.034 | 0.282 | Yes | Segmental duplication |
|  | *GhSIP1;2_D* | *GhSIP1;2_A* | 96.900 | 0.022 | 0.084 | 0.267 | Yes | Segmental duplication |
|  | *GhXIP1;2_D* | *GhXIP1;2_A* | 98.250 | 0.010 | 0.040 | 0.257 | Yes | Segmental duplication |
|  | *GhNIP1;1_D* | *GhNIP1;1_A* | 96.920 | 0.018 | 0.075 | 0.240 | Yes | Segmental duplication |
|  | *GhPIP1;2_A* | *GhPIP1;1_D* | 91.800 | 0.125 | 0.552 | 0.227 | Yes | Segmental duplication |
|  | *GhPIP1;2_A* | *GhPIP1;1_A* | 91.430 | 0.130 | 0.587 | 0.221 | Yes | Segmental duplication |
|  | *GhPIP2;2_D* | *GhPIP2;2_A* | 99.430 | 0.003 | 0.014 | 0.218 | Yes | Segmental duplication |
|  | *GhPIP2;6_D* | *GhPIP2;1_A* | 99.420 | 0.004 | 0.017 | 0.215 | Yes | Segmental duplication |
|  | *GhPIP2;6_A* | *GhPIP2;1_A* | 98.830 | 0.006 | 0.029 | 0.211 | Yes | Tandem duplication |
|  | *GhTIP4;1_D* | *GhTIP4;1_A* | 98.720 | 0.011 | 0.055 | 0.206 | Yes | Segmental duplication |
|  | *GhTIP1;4_D* | *GhTIP1;4_A* | 97.500 | 0.012 | 0.066 | 0.189 | Yes | Segmental duplication |
|  | *GhNIP7;1_D* | *GhNIP7;1_A* | 99.050 | 0.004 | 0.023 | 0.188 | Yes | Segmental duplication |
|  | *GhPIP2;10_D* | *GhPIP2;10_A* | 99.280 | 0.003 | 0.019 | 0.168 | Yes | Segmental duplication |
|  | *GhPIP2;11_D* | *GhPIP2;11_A* | 98.920 | 0.005 | 0.030 | 0.162 | Yes | Segmental duplication |
|  | *GhPIP2;7_D* | *GhPIP2;7_A* | 99.300 | 0.003 | 0.019 | 0.160 | Yes | Segmental duplication |
|  | *GhPIP2;9_D* | *GhPIP2;9_A* | 97.880 | 0.009 | 0.060 | 0.159 | Yes | Segmental duplication |
|  | *GhPIP1;9_D* | *GhPIP1;9_A* | 96.530 | 0.006 | 0.045 | 0.142 | Yes | Segmental duplication |
|  | *GhPIP1;10_D* | *GhPIP1;10_A* | 98.840 | 0.005 | 0.033 | 0.140 | Yes | Segmental duplication |
|  | *GhNIP6;1_D* | *GhNIP6;1_A* | 98.730 | 0.005 | 0.036 | 0.137 | Yes | Segmental duplication |
|  | *GhNIP3;1_D* | *GhNIP3;1_A* | 99.010 | 0.005 | 0.033 | 0.137 | Yes | Segmental duplication |
|  | *GhPIP2;5_A* | *GhPIP2;12_A* | 90.220 | 0.188 | 1.420 | 0.132 | Yes | Segmental duplication |
|  | *GhPIP1;7_A* | *GhPIP1;4_D* | 99.200 | 0.003 | 0.024 | 0.128 | Yes | Segmental duplication |
|  | *GhTIP1;7_A* | *GhTIP1;6_D* | 92.940 | 0.055 | 0.428 | 0.128 | Yes | Segmental duplication |
|  | *GhPIP2;5_D* | *GhPIP2;12_A* | 93.510 | 0.184 | 1.449 | 0.127 | Yes | Segmental duplication |
|  | *GhTIP1;7_D* | *GhTIP1;6_D* | 92.570 | 0.053 | 0.436 | 0.121 | Yes | Segmental duplication |
|  | *GhPIP2;5_A* | *GhPIP2;12_D* | 90.220 | 0.187 | 1.572 | 0.119 | Yes | Segmental duplication |
|  | *GhTIP2;2_D* | *GhTIP2;2_A* | 98.540 | 0.004 | 0.031 | 0.117 | Yes | Segmental duplication |
|  | *GhTIP2;1_D* | *GhTIP2;1_A* | 98.930 | 0.004 | 0.031 | 0.116 | Yes | Segmental duplication |
|  | *GhXIP1;1_D* | *GhXIP1;1_A* | 98.250 | 0.006 | 0.052 | 0.115 | Yes | Segmental duplication |
|  | *GhPIP2;5_D* | *GhPIP2;12_D* | 93.510 | 0.183 | 1.608 | 0.114 | Yes | Segmental duplication |
|  | *GhNIP1;2_D* | *GhNIP1;2_A* | 99.030 | 0.003 | 0.029 | 0.111 | Yes | Segmental duplication |
|  | *GhPIP1;7_D* | *GhPIP1;6_A* | 98.620 | 0.005 | 0.044 | 0.106 | Yes | Segmental duplication |
|  | *GhTIP1;5_D* | *GhTIP1;5_A* | 97.760 | 0.007 | 0.071 | 0.101 | Yes | Segmental duplication |
|  | *GhPIP1;9_D* | *GhPIP1;2_A* | 91.210 | 0.194 | 1.949 | 0.100 | Yes | Segmental duplication |
|  | *GhPIP2;7_A* | *GhPIP2;2_A* | 90.000 | 0.108 | 1.118 | 0.097 | Yes | Segmental duplication |
|  | *GhPIP2;12_D* | *GhPIP2;12_A* | 98.450 | 0.005 | 0.050 | 0.096 | Yes | Segmental duplication |
|  | *GhSIP1;5_D* | *GhSIP1;5_A* | 98.090 | 0.005 | 0.063 | 0.087 | Yes | Segmental duplication |
|  | *GhPIP2;8_D* | *GhPIP2;8_A* | 99.420 | 0.002 | 0.019 | 0.081 | Yes | Segmental duplication |
|  | *GhPIP2;9_D* | *GhPIP2;11_D* | 91.090 | 0.142 | 1.781 | 0.080 | Yes | Segmental duplication |
|  | *GhPIP1;1_D* | *GhPIP1;1_A* | 98.840 | 0.003 | 0.039 | 0.079 | Yes | Segmental duplication |
|  | *GhPIP1;5_D* | *GhPIP1;4_A* | 98.380 | 0.005 | 0.058 | 0.079 | Yes | Segmental duplication |
|  | *GhPIP2;8_D* | *GhPIP2;7_D* | 90.400 | 0.107 | 1.361 | 0.078 | Yes | Segmental duplication |
|  | *GhPIP2;9_A* | *GhPIP2;11_A* | 90.820 | 0.140 | 1.817 | 0.077 | Yes | Segmental duplication |
|  | *GhPIP2;9_D* | *GhPIP2;11_A* | 90.820 | 0.141 | 1.965 | 0.072 | Yes | Segmental duplication |
|  | *GhNIP7;2_D* | *GhNIP7;2_A* | 98.750 | 0.002 | 0.035 | 0.066 | Yes | Segmental duplication |
|  | *GhPIP2;4_D* | *GhPIP2;4_A* | 99.290 | 0.002 | 0.024 | 0.066 | Yes | Segmental duplication |
|  | *GhPIP1;9_A* | *GhPIP1;10_A* | 90.230 | 0.027 | 0.412 | 0.065 | Yes | Segmental duplication |
|  | *GhPIP2;5_D* | *GhPIP2;5_A* | 98.600 | 0.003 | 0.049 | 0.064 | Yes | Segmental duplication |
|  | *GhTIP3;1_D* | *GhTIP3;1_A* | 99.090 | 0.002 | 0.030 | 0.059 | Yes | Segmental duplication |
|  | *GhPIP1;6_D* | *GhPIP1;5_D* | 98.610 | 0.003 | 0.053 | 0.058 | Yes | Tandem duplication |
|  | *GhTIP1;3_D* | *GhTIP1;3_A* | 98.510 | 0.004 | 0.064 | 0.057 | Yes | Segmental duplication |
|  | *GhTIP1;2_D* | *GhTIP1;2_A* | 98.150 | 0.004 | 0.065 | 0.056 | Yes | Segmental duplication |
|  | *GhPIP1;7_D* | *GhPIP1;5_D* | 96.290 | 0.008 | 0.144 | 0.054 | Yes | Tandem duplication |
|  | *GhTIP1;7_A* | *GhTIP1;6_A* | 90.500 | 0.022 | 0.417 | 0.052 | Yes | Segmental duplication |
|  | *GhPIP1;5_D* | *GhPIP1;5_A* | 97.680 | 0.005 | 0.089 | 0.052 | Yes | Segmental duplication |
|  | *GhPIP1;7_D* | *GhPIP1;4_A* | 96.660 | 0.006 | 0.127 | 0.048 | Yes | Segmental duplication |
|  | *GhPIP1;2_D* | *GhPIP1;1_A* | 90.630 | 0.020 | 0.421 | 0.048 | Yes | Segmental duplication |
|  | *GhPIP1;7_D* | *GhPIP1;5_A* | 96.540 | 0.006 | 0.133 | 0.046 | Yes | Segmental duplication |
|  | *GhPIP1;6_D* | *GhPIP1;4_A* | 99.080 | 0.002 | 0.033 | 0.046 | Yes | Segmental duplication |
|  | *GhTIP1;7_D* | *GhTIP1;6_A* | 90.360 | 0.020 | 0.434 | 0.046 | Yes | Segmental duplication |
|  | *GhPIP1;6_A* | *GhPIP1;5_D* | 96.410 | 0.006 | 0.144 | 0.043 | Yes | Segmental duplication |
|  | *GhPIP1;2_D* | *GhPIP1;1_D* | 91.090 | 0.016 | 0.408 | 0.040 | Yes | Segmental duplication |
|  | *GhPIP1;7_D* | *GhPIP1;6_D* | 96.890 | 0.005 | 0.122 | 0.038 | Yes | Tandem duplication |
|  | *GhNIP5;1_D* | *GhNIP5;1_A* | 98.660 | 0.002 | 0.047 | 0.032 | Yes | Segmental duplication |
|  | *GhPIP1;6_D* | *GhPIP1;6_A* | 96.890 | 0.003 | 0.128 | 0.024 | Yes | Segmental duplication |
|  | *GhPIP1;6_D* | *GhPIP1;5_A* | 97.580 | 0.002 | 0.100 | 0.015 | Yes | Segmental duplication |
|  | *GhPIP1;6_A* | *GhPIP1;4_A* | 97.350 | 0.002 | 0.111 | 0.014 | Yes | Tandem duplication |
|  | *GhPIP1;6_A* | *GhPIP1;5_A* | 97.230 | 0.002 | 0.116 | 0.013 | Yes | Tandem duplication |
|  | *GhTIP1;7_D* | *GhTIP1;7_A* | 99.080 | 0.000 | 0.037 | 0.000 | Yes | Segmental duplication |
|  | *GhTIP1;1_D* | *GhTIP1;1_A* | 99.690 | 0.000 | 0.058 | 0.000 | Yes | Segmental duplication |
|  | *GhPIP1;5_A* | *GhPIP1;4_A* | 99.780 | 0.000 | 0.095 | 0.000 | Yes | Tandem duplication |
| **A_2_** | *GaSIP1;4* | *GaSIP1;3* | 96.540 | 0.055 | 0.125 | 0.442 | Yes | Segmental duplication |
|  | *GaPIP2;3* | *GaPIP2;12* | 90.540 | 0.145 | 1.351 | 0.108 | Yes | Segmental duplication |
|  | *GaPIP2;7* | *GaPIP2;2* | 90.000 | 0.104 | 1.111 | 0.094 | Yes | Segmental duplication |
|  | *GaPIP2;12* | *GaPIP2;1* | 90.540 | 0.139 | 1.490 | 0.093 | Yes | Segmental duplication |
|  | *GaPIP2;4* | *GaPIP2;11* | 90.820 | 0.140 | 1.811 | 0.078 | Yes | Segmental duplication |
|  | *GaPIP1;6* | *GaPIP1;3* | 90.540 | 0.082 | 1.116 | 0.074 | Yes | Segmental duplication |
|  | *GaPIP1;9* | *GaPIP1;10* | 90.230 | 0.028 | 0.408 | 0.067 | Yes | Segmental duplication |
|  | *GaTIP1;7* | *GaTIP1;6* | 90.090 | 0.022 | 0.444 | 0.049 | Yes | Segmental duplication |
|  | *GaPIP1;2* | *GaPIP1;1* | 91.090 | 0.019 | 0.396 | 0.047 | Yes | Segmental duplication |
|  | *GaPIP1;6* | *GaPIP1;4* | 97.230 | 0.002 | 0.117 | 0.013 | Yes | Tandem duplication |
| **D_5_** | *GrPIP1;5* | *GrPIP1;6* | 95.870 | 0.002 | 0.048 | 0.032 | Yes | Tandem duplication |
|  | *GrPIP2;4* | *GrPIP2;5* | 98.100 | 0.011 | 0.050 | 0.222 | Yes | Tandem duplication |
|  | *GrPIP1;6* | *GrPIP1;7* | 91.300 | 0.005 | 0.144 | 0.032 | Yes | Tandem duplication |
|  | *GrPIP1;5* | *GrPIP1;7* | 90.830 | 0.006 | 0.144 | 0.043 | Yes | Tandem duplication |
|  | *GrSIP1;3* | *GrSIP1;4* | 93.550 | 0.032 | 0.076 | 0.418 | Yes | Segmental duplication |
|  | *GrPIP2;7* | *GrPIP2;12* | 90.230 | 0.229 | 1.859 | 0.123 | Yes | Segmental duplication |
|  | *GrPIP1;1* | *GrPIP1;2* | 91.090 | 0.016 | 0.384 | 0.043 | Yes | Segmental duplication |
|  | *GrPIP2;3* | *GrPIP2;12* | 90.400 | 0.257 | 1.461 | 0.176 | Yes | Segmental duplication |
|  | *GrPIP2;9* | *GrPIP2;11* | 90.630 | 0.144 | 1.973 | 0.073 | Yes | Segmental duplication |
|  | *GrPIP2;10* | *GrPIP2;12* | 96.310 | 0.128 | 0.959 | 0.133 | Yes | Segmental duplication |
|  | *GrTIP1;4* | *GrTIP1;5* | 91.440 | 0.020 | 0.416 | 0.048 | Yes | Segmental duplication |
|  | *GrPIP2;2* | *GrPIP2;8* | 96.420 | 0.102 | 1.409 | 0.072 | Yes | Segmental duplication |
|  | *GrPIP1;9* | *GrPIP1;10* | 98.730 | 0.031 | 0.391 | 0.079 | Yes | Segmental duplication |

AD_1_, *G. hirsutum*; A_2_, *G. arboretum*; D_5_, *G. raimondii.*

**MATERIALS AND METHODS**

**Data mining, AQPs identification and phylogenetic analysis in *Gossypium* Spp**

Genome databases of *G. arboreum* (A_2_, BGI_version 1.0), *G. raimondii* (D_5_, JGI_version 2.1), and *G. hirsutum* acc. TM-1 (AD_1_, NBI_version 1.1) was obtained from the CottonGen website (Yu et al., 2014). The published data on aquaporins of *Arabidopsis* were obtained from TAIR (<http://www.arabidopsis.org/>). Aquaporin sequences of *Oryza sativa* and *Phaseolus vulgaris* were obtained from the Rice Genome Annotation Project (RGAP) databases (<http://rice.plantbiology.msu.edu/>) and Phytozome (<http://phytozome.jgi.doe.gov/pz/portal.html>). For ease of description, *G. raimondii, G. arboreum, G. hirsutum,* *A. thaliana, O. sativa,* and *P. vulgaris* were hereafter abbreviated as Gr, Ga, Gh, At, Os, and Pv, respectively. The aquaporin genes were extracted from the whole genome sequences of the above three cotton species, and the MIP domain (Pfam accessions, PF00230) was used to build Hidden Markov Model (HMM) for searching AQPs in *Gossypium* using the HMMER program (Finn et al., 2011).

The amino acid sequences of aquaporins in three cotton species, *O. sativa*, *P. vulgaris,* and *Arabidopsis* were used for multiple sequence alignment. The Maximum Likelihood method was applied to construct a phylogenetic tree and the reliability was obtained by bootstrapping with 1000 replicates using software MEGA 8.

**Structural features and promoter analysis of AQPs**

The exon-intron structure was performed by Gene Structure Display Server (GSDS) tool (http://gsds.cbi.pku.edu.cn/) based on genes coding sequences and the annotated genome.

Protein sequences of AQP in *G. hirsutum* were submitted to an online MEME program to identify the top ten conserved motifs with default parameters (Bailey et al., 2009).

The 1500 bp upstream sequences before the initiation codon (ATG) of *AQPs* in *G. hirsutum* were considered as promotors. Cis-regulatory elements of each promoter sequence were predicted against the PlantCARE database (<http://bioinformatics.psb.ugent.be/webtools/plantcare/html/>). The cis-regulatory elements in the promoter were visualized by TBtools software (<http://cj-chen.github.io/tbtools/>).

**Chromosomal location and gene duplication analysis**

The chromosomal localization of aquaporin genes was retrieved from the CottonGen website (https://www.cottongen.org/). Mapchart Version 2.3 software was used to visualize the distribution of the aquaporin genes on the chromosomes.

Gene duplication events were defined when the following conditions were fulfilled: (1) the length of aligned sequence covered more than 75% of the larger gene, (2) the identity of the aligned regions are bigger than 75%, and (3) only one duplication event was considered for tightly linked genes (Cui et al., 2017). According to the chromosomal locations of aquaporin genes, two types of gene duplication events (tandem duplication and segmental duplication) were recognized (Vision et al., 2000). The duplicated gene pairs were aligned by Clustal X program. Subsequently, non-synonymous substitution (*Ka*) and synonymous substitution (*Ks*) were calculated using DnaSP software version 5.5, and the selection pressure for each gene pair was assessed by the *Ka/Ks* ratio.
