# Supplementary figures and images for: Two Aquaporin Genes, GhPIP2;7 and GhTIP2;1, Positively Regulate the Tolerance of Upland Cotton to Salt and Osmotic Stresses
Source: Front Plant Sci. 2022 Feb 11;12:780486. doi: 10.3389/fpls.2021.780486 (PMC8873789; doi:10.3389/fpls.2021.780486)

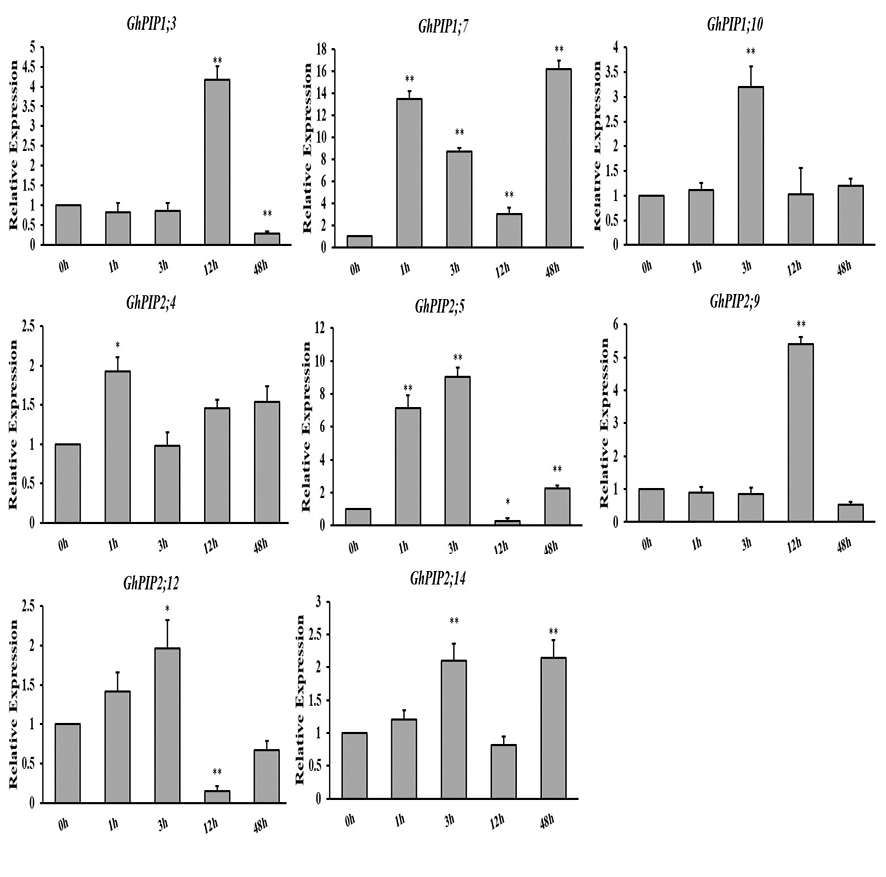

Supplement: Supplementary Figure S1 — Expression patterns of candidate genes under salt stress. The expression patterns of candidate genes under salt stress (150 mM). Quantitative RT-PCR was used to investigate the expression levels of candidate genes. GhUBQ7 was used as the internal control to calculate and normalize the expression levels. [file Image_1.TIF]

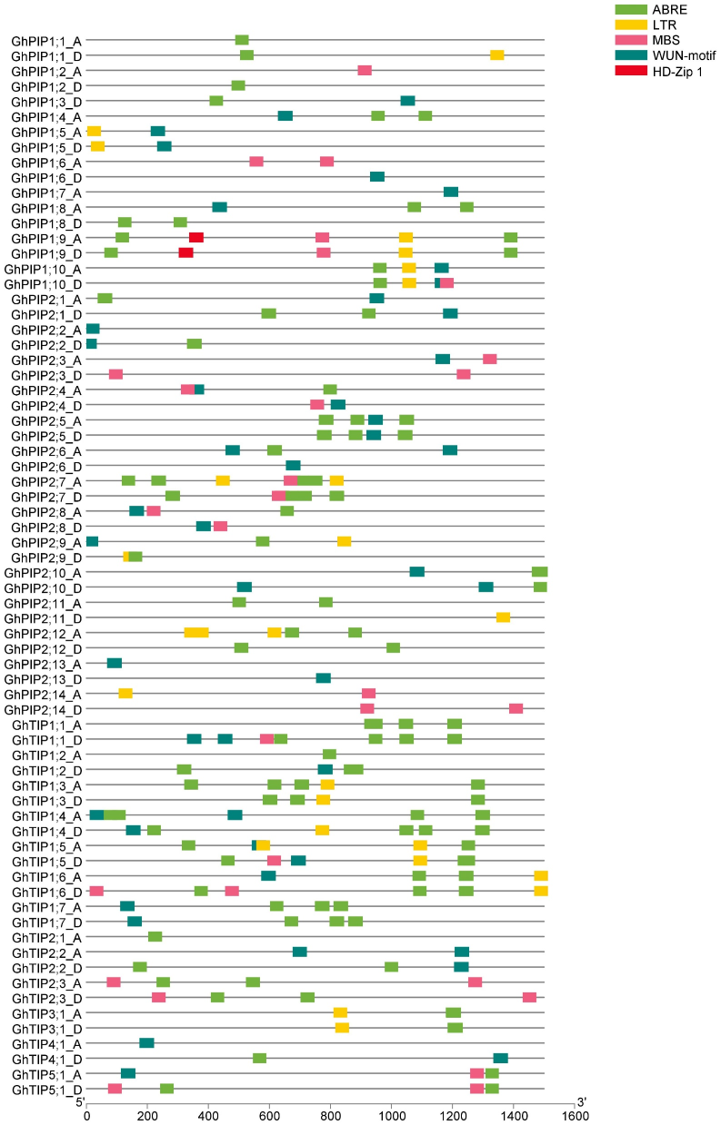

Supplement: Supplementary Figure S2 — Predicted cis-elements respond to abiotic stress in promoter regions of GhAQPs. ARE, a cis-acting regulatory element essential for the anaerobic induction; LTR, a cis-acting element involved in low-temperature responsiveness; MBS, MYB-binding site involved in drought inducibility; HD-Zip 1, motif involved in the differentiation of the palisade mesophyll cells, WUN-motif, wound-responsive element. [file Image_2.TIFF]

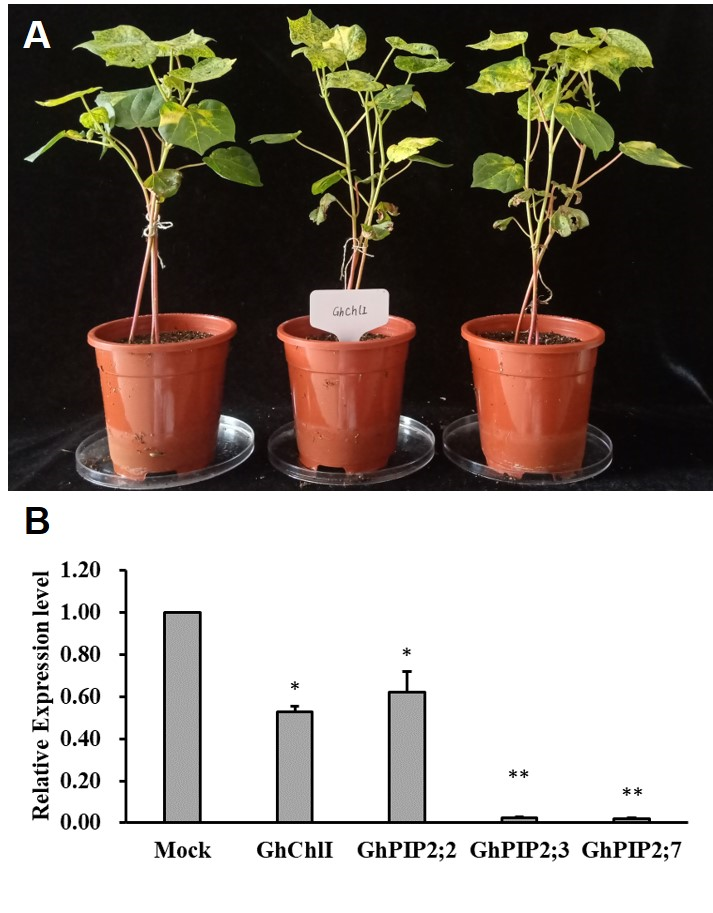

Supplement: Supplementary Figure S3 — Silencing efficiency and phenotype identification of GhPIP2;2-, GhPIP2;3-, GhPIP2;7-, and GhChlI-silenced plants in cotton. (A) The phenotype of cotton plants infiltrated with the CLCrV-ChlI-vector (positive control) after 14 days. (B) The relative expression level of the target gene in cotton infiltrated with the CLCrV-based empty vector (mock), CLCrV- ChlI (positive control), CLCrV-GhPIP2;2, CLCrV-GhPIP2;3, and CLCrV- GhPIP2;7 vectors. **Represents that the expression of the gene was significantly different from that of the control. Data are the mean of three replications ± SE. [file Image_3.TIFF]
